# Photoinduced Electron Transfer in Multicomponent Truxene-Quinoxaline Metal–Organic Frameworks

**DOI:** 10.1021/acs.chemmater.2c02220

**Published:** 2022-09-15

**Authors:** Joel Cornelio, Seok June Lee, Tian-You Zhou, Adil Alkaş, Kavipriya Thangavel, Andreas Pöppl, Shane G. Telfer

**Affiliations:** † School of Natural Sciences, MacDiarmid Institute of Advanced Materials and Nanotechnology, Massey University, Palmerston North 4410, New Zealand; ‡ Felix Bloch Institute for Solid State Physics, Leipzig University, Linnestrasse 5, Leipzig D-04103, Germany

## Abstract

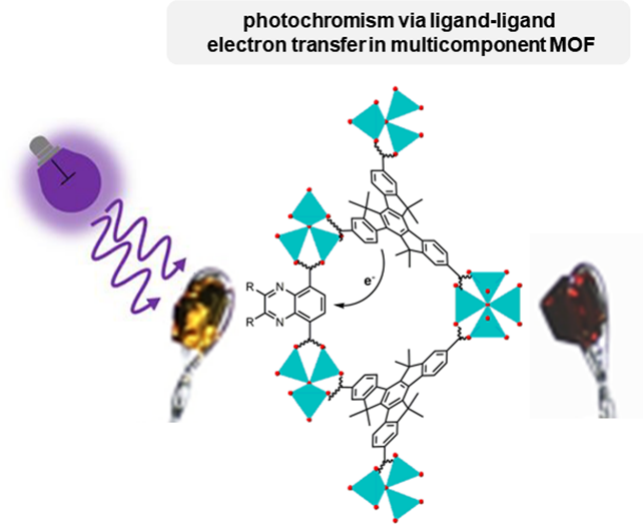

Metal–organic frameworks (MOFs) can respond to light in a number of interesting ways. Photochromism is observed when a structural change to the framework is induced by the absorption of light, which results in a color change. In this work, we show that introducing quinoxaline ligands to MUF-7 and MUF-77 (MUF = Massey University Framework) produces photochromic MOFs that change color from yellow to red upon the absorption of 405 nm light. This photochromism is observed only when the quinoxaline units are incorporated into the framework and not for the standalone ligands in the solid state. Electron paramagnetic resonance (EPR) spectroscopy shows that organic radicals form upon irradiation of the MOFs. The EPR signal intensities and longevity depend on the precise structural details of the ligand and framework. The photogenerated radicals are stable for long periods in the dark but can be switched back to the diamagnetic state by exposure to visible light. Single-crystal X-ray diffraction analysis reveals bond length changes upon irradiation that are consistent with electron transfer. The multicomponent nature of these frameworks allows the photochromism to emerge by allowing through-space electron transfer, precisely positioning the framework building blocks, and tolerating functional group modifications to the ligands.

## Introduction

Metal–organic frameworks (MOFs) can exhibit interesting behavior in response to external physical or chemical stimuli. Frameworks where a reversible structural change can be induced are of particular interest since they may find use as molecular switches.^1,2^ Relevant stimuli include the adsorption or expulsion of guests^3,4^ and fluctuations in temperature^5^ and pressure.^6^ Photochromism is a phenomenon in which the color of a material changes on exposure to light. Here, the absorption of light stimulates a structural change that results in a color change.^7^ The color change can often be reversed thermally or by photons of a different wavelength. Although photochromism has been observed in MOFs, it is restricted to a few families of ligands such as spiropyrans, azobenzenes, napthalenediimides, and diarylethenes.^8−12^ In all these cases, the ligands are photochromic even when they are in standalone form. Their photochromism carries over when they are used to prepare MOFs. There are only a few examples of nonphotochromic ligands becoming photochromic upon incorporation into a MOF;^13^ however, such behavior is more common for discrete transition metal complexes and supramolecular assemblies.^12,14,15^

While radical-bearing MOFs have been extensively detailed, most of them feature the radical as a guest in the framework pores.^16^ Here, the pore environment typically stabilizes the radical, which would otherwise be fairly short-lived. Alternatively, the framework ligand can be a radical and, thus, the radical is key to the structural integrity of the MOF.^17−19^ A third type of radical-bearing MOF is most relevant to the current work. Here, a framework ligand generates a radical in response to the absorption of light, which leads to photoinduced electron transfer.^20−22^ Since the radical and nonradical structures often have different colors, the formation of radicals in this way is generally associated with photochromism.^23^

**Figure 1:**
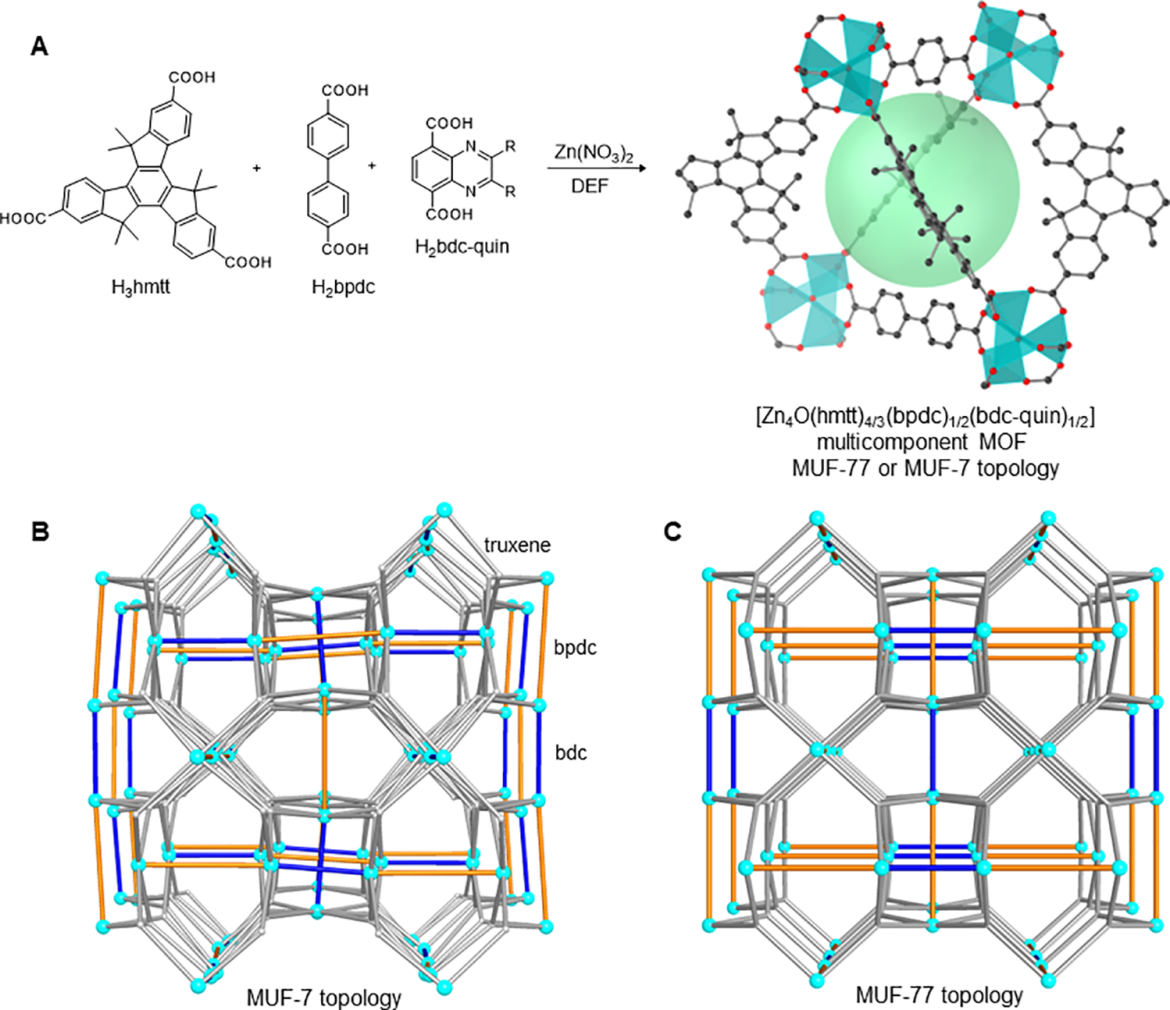
(A) Synthesis of multicomponent [Zn_4_O(hmtt)_4/3_(bpdc)_1/2_(bdc)_1/2_] MOFs with bdc-quin ligands. The frameworks adopt either the MUF-7 or MUF-77 topology. Cyan = Zn; black = C; red = O; green sphere = void space. H atoms and quinoxaline group omitted for clarity. (B) MUF-7 framework topology. The tritopic truxene linkers are shown as gray rods, the bpdc linkers as orange rods, the bdc linkers as blue rods, and the centers of the Zn_4_O clusters as cyan spheres. (C) MUF-77 framework topology.

Since photoinduced electron transfer in MOFs typically involves two distinct framework components, we sought to develop radical-based photochromic properties in MUF-7 and MUF-77 (MUF = Massey University Framework).^24−30^ MUF-7 and MUF-77 are multicomponent MOFs that comprise three different carboxylate ligands linked together by Zn_4_O clusters. The ligand set comprises a tritopic tris(carboxylate) linker based on a truxene core, such as hexamethyltruyxenetricarboxylic acid (H_3_hmtt), and ditopic linkers based on the 4,4′-biphenyldicarboxylic acid (H_2_bpdc) and 1,4-benzenedicarboxylic acid (H_2_bdc) backbones (Figure 1A). The three ligands occupy distinct positions in both the MUF-7 and MUF-77 frameworks. Various functional groups can be appended to the three ligands without perturbing the global structure of the network.

The formulae of MUF-7 and MUF-77 are the same, [Zn_4_O(hmtt)_4/3_(bpdc)_1/2_(bdc)_1/2_], but they are topological isomers of one another and differ subtly in the arrangement of the constituent ligands (Figure 1B,C). They can be distinguished crystallographically: while both are cubic, MUF-7 has a unit cell parameter around 60 Å and belongs to the *I*-43*d* space group, while MUF-77 has a unit cell parameter around 30 Å and belongs to the *Pm*-3 space group.^25,26,29,30^ In their powder X-ray diffraction (PXRD) patterns, MUF-7 has a shoulder at 2θ = 3.5° and a peak at 5.5°, while MUF-77 has a peak at 2θ = 3.0°, 5.1°, and 6.1° (Cu_α_ radiation). It is important to note that for this work, the substituents on the bdc linker determined whether the MUF-7 or MUF-77 topology is produced.

## Experimental Section

### General Procedure for the Synthesis of Quinoxaline Esters

In a typical synthesis, compound **5** (1 equiv) was combined with the diketone (1.1 equiv). Nine milliliters of 1:2 EtOH/AcOH was added, and the mixture was heated on reflux for 3 h (Scheme S2). Anhydrous MgSO_4_ was added, and the refluxing was continued for an additional 12 h. The reaction mixture was added to water dropwise to precipitate out the crude product, which was filtered. The residue was washed with 1:1 hexane:DCM (this fraction was rejected) followed by a wash with pure DCM and MeOH, which was collected and dried to give compounds **6**, **7**, **8**, **9**, and **10**.

### General Procedure for the Synthesis of H_2_bdc-quin Ligands

For obtaining quinoxaline carboxylic acid ligands by hydrolysis, the diethylesters (**6**, **7**, **8**, **9**, or **10**) were dissolved in 20 mL of 1:1 THF:2 M aq. KOH and heated at 45 °C overnight (Scheme S3). THF was removed using a separating funnel, and the aqueous layer was neutralized using 2 M HCl to a pH of 3–4 to precipitate out the carboxylic acids. The solid obtained was filtered, washed with water, and dried to give ligands H_2_bdc-dpq, H_2_bdc-dpq-OMe, H_2_bdc-paq, H_2_bdc-paq-Br, and H_2_bdc-thenil.

### Synthesis of Photochromic MUF-7 and MUF-77 Single Crystals

H_3_hmtt, H_2_bpdc, and H_2_bdc-quin ligand (details in Scheme S4 and Table S1) were dissolved in 1 mL of anhy. *N*,*N*-diethylformamide, and 50 μL of water was added. Zinc nitrate tetrahydrate was added, and the sample was sonicated briefly and placed in an isothermal oven kept at 85 °C for 24 h. The mother liquor was decanted when hot, and the crystals were washed with anhydrous DMF and stored in DMF at room temperature.

### Synthesis of Nanocrystalline, Photochromic MUF-7 and MUF-77

H_3_hmtt, H_2_bpdc, and H_2_bdc-quinoxaline ligands (details in Scheme S5 and Table S2) were stirred and dissolved in 2.5 mL of a 50:1 v/v solution of *N*,*N*-dimethylformamide:water. Solid Zn(OAc)_2_·2H_2_O was added, and the stirring was continued for another 30 min. The resulting suspension was centrifuged thrice with fresh DMF, suspended in DMF, and placed in an isothermal oven at 85 °C overnight. The crystals were centrifuged again with fresh, dry DMF and stored at room temperature.

## Results and Discussion

### Synthesis and Characterization of Photochromic Multicomponent MOFs

**Figure 2:**
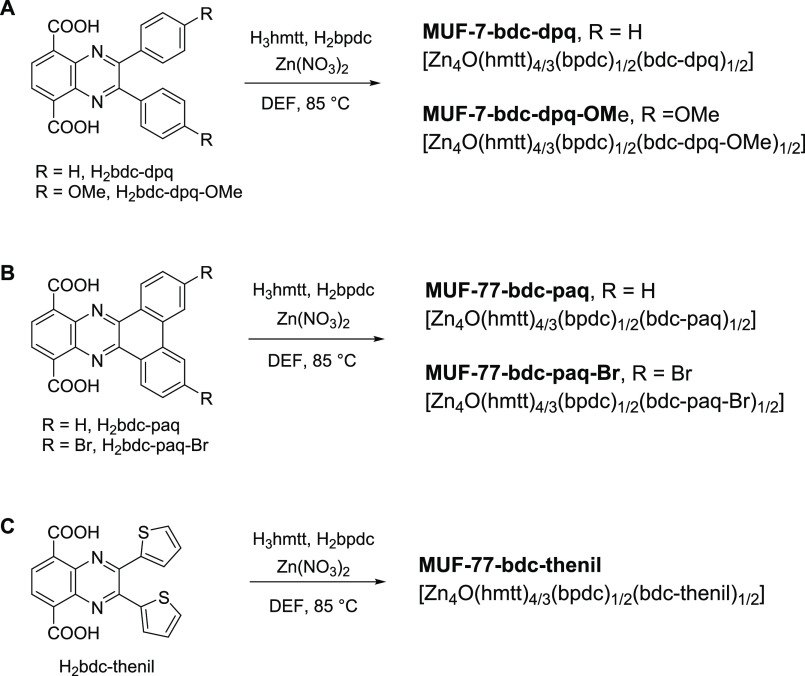
Synthetic routes to photochromic MUF-7 and MUF-77 frameworks containing the bdc-quin ligands bdc-dpq, bdc-dpq-OMe, bdc-paq, bdc-paq-Br, and bdc-thenil.

Pyrazine derivatives of the bdc linker were synthesized in the form of quinoxalines using a multistep procedure (Schemes S1–S3). The synthetic route was designed to be amenable to the late-stage incorporation of a variety of substituents on the pyrazine ring. The resulting ligands, H_2_bdc-dpq, H_2_bdc-dpq-OMe, H_2_bdc-paq, H_2_bdc-thenil, and H_2_bdc-paq-Br, are shown in Figure 2 and collectively referred to as H_2_bdc-quin.

**Figure 3:**
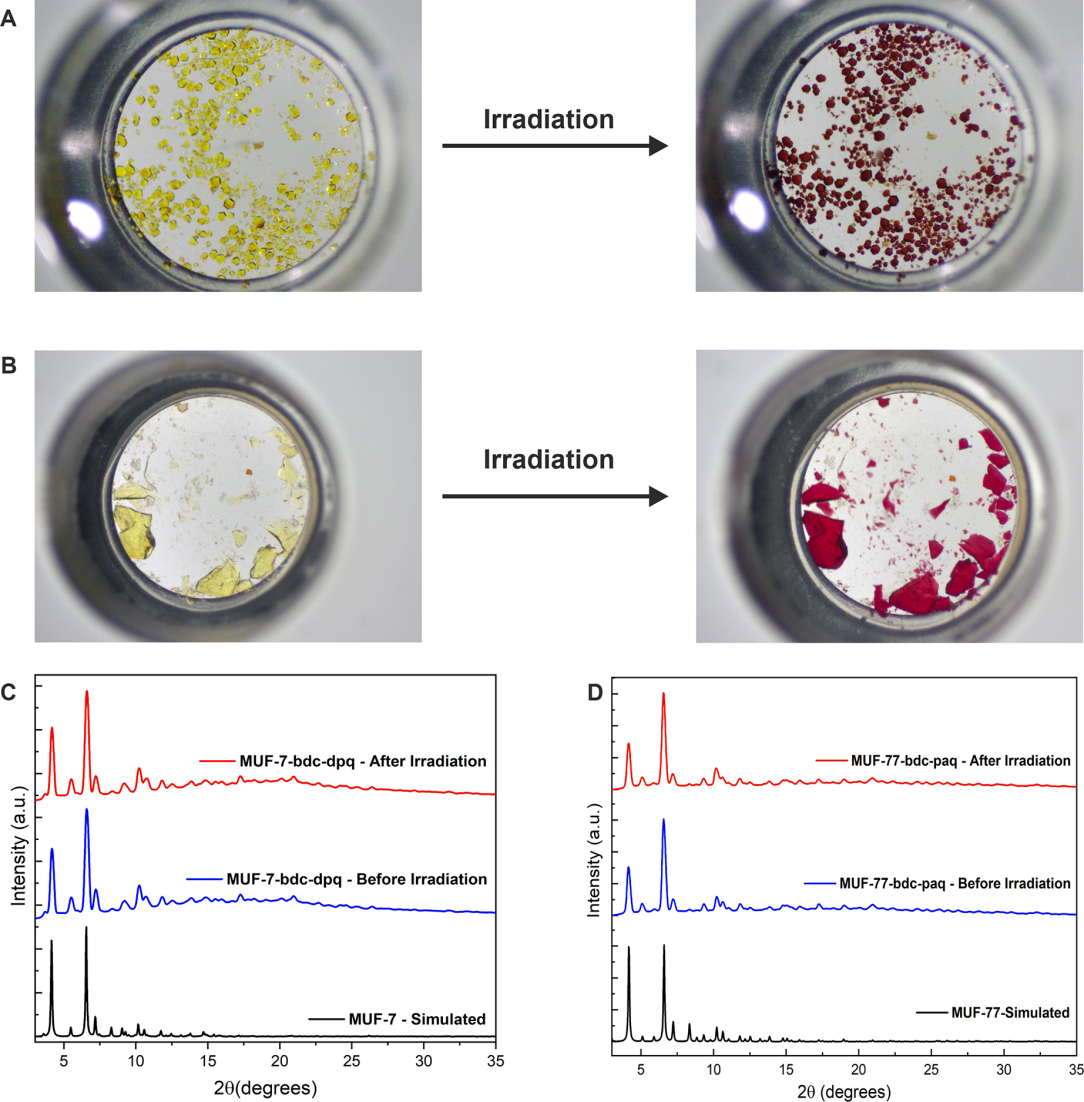
(A) Photos of MUF-7-bdc-dpq-OMe before and after irradiation with 405 nm light. (B) Photos of MUF-77-bdc-paq before and after irradiation with 405 nm light. Representative PXRD (Cu_α_ radiation) patterns of (C) photochromic MUF-7-bdc-dpq and (D) MUF-77-bdc-paq crystals before and after irradiation. The PXRD patterns simulated from the SCXRD structures are provided for comparison.^29,30^

The solvothermal synthesis of H_3_hmtt, H_2_bpdc, H_2_bdc-dpq, and zinc nitrate in *N*,*N*-diethylformamide (DEF) yields pale yellow cubic crystals of [Zn_4_O(hmtt)_4/3_(bpdc)_1/2_(bdc-dpq)_1/2_] (Figure 2A). PXRD revealed that the framework adopts the MUF-7 structure (Figure 3A). This framework isomer was retained when H_2_bdc-dpq was replaced by H_2_bdc-dpq-OMe, giving crystals with the formula [Zn_4_O(hmtt)_4/3_(bpdc)_1/2_(bdc-dpq-OMe)_1/2_] (PXRD in Figure S2). We also determined the unit cell parameters of these crystals by single-crystal X-ray diffraction (SCXRD). The cells belonged to a cubic system with unit cell lengths closely matching that of other MUF-7 systems (Table 1). ^1^H NMR spectroscopy on digested frameworks (Figures S25 and S26) allowed the ratio of the three ligands to be assessed, which was in accord with the formulae of MUF-7-bdc-dpq and MUF-7-bdc-dpq-OMe.

**Table 1:** Crystallographic Details for the Photochromic Multicomponent MOFs

framework	ligand combination	unit cell length (*a* = *b* = *c*), Å	framework isomer
MUF-7-bdc-dpq	hmtt/bpdc/bdc-dpq	59.5745(16)	MUF-7
MUF-7-bdc-dpq-OMe	hmtt/bpdc/bdc-dpq-OMe	59.8236(17)	MUF-7
MUF-77-bdc-paq	hmtt/bpdc/bdc-paq	29.8935(12)	MUF-77
MUF-77-bdc-paq-Br	hmtt/bpdc/bdc-paq-Br	29.9583(5)	MUF-77
MUF-77-bdc-thenil	hmtt/bpdc/bdc-thenil	29.9673(5)	MUF-77

The solvothermal synthesis of zinc nitrate, H_3_hmtt and H_2_bpdc, and one of H_2_bdc-paq, H_2_bdc-paq-Br, and H_2_bdc-thenil in DEF also produced pale yellow cubic crystals (Figure 2B,C and Scheme S4). X-ray diffraction analysis showed that these frameworks adopted the MUF-77 structure: their PXRD patterns were consistent with MUF-77 and the unit cell dimension was 29.9 Å (Table 1, Figure S3, and Table S7). These MOFs are referred to as MUF-77-bdc-paq, MUF-77-bdc-paq-Br, and MUF-77-bdc-thenil (Table 1). ^1^H NMR spectroscopy on digested samples showed the formula to be [Zn_4_O(hmtt)_4/3_(bpdc)_1/2_(bdc-quin)_1/2_], as expected (Figures S27–S29).

The five MOFs listed in Table 1 are photochromic. Upon exposing them to 405 nm irradiation using a UV laser pointer (1 mW), their color changed from yellow to red over a few seconds (Figure 3A,B). The crystal color reverted to yellow in a few minutes once the irradiation was stopped. This process could be repeated indefinitely. PXRD patterns recorded before and after light exposure were identical (Figure 3C,D), indicating that the global framework structure was retained upon irradiation. However, placing these materials in the dark after irradiation prolongs the duration over which the red color persists. This indicates that the reverse process is triggered photochemically by visible light and not thermally.

To make the crystals more amenable to spectroscopic measurements, we synthesized nanocrystalline versions of the photochromic MOFs listed in Table 1 by using zinc acetate instead of zinc nitrate and performing the synthesis at room temperature (Scheme S5 and Table S2).^27,31^ These materials are referred to as NC-MUF-7-*x* and NC-MUF-77-*x*. PXRD and ^1^H NMR spectroscopy on digested samples confirmed that MUF-7 and MUF-77 were formed in each case, and the framework isomer observed by SCXRD was retained by the bulk materials (Figures S4, S5, and S30–S34). The nanocrystalline materials also showed yellow-to-red photochromism upon exposure to a 405 nm laser pointer (Figure S1). Again, their PXRD patterns remained unchanged during this process (Figures S4 and S5).

Photochromism was observed in the NC-MOFs upon exposure to direct sunlight. A yellow-to-red color change was seen for all materials in about 5 min, although the depth of the red coloration depended on the sets of ligands in the framework (Figure S7). NC-MUF-77-thenil showed the least amount of color change, while the red color of NC-MUF-77-bdc-paq and NC-MUF-7-bdc-dpq-OMe was very pronounced.

**Figure 4:**
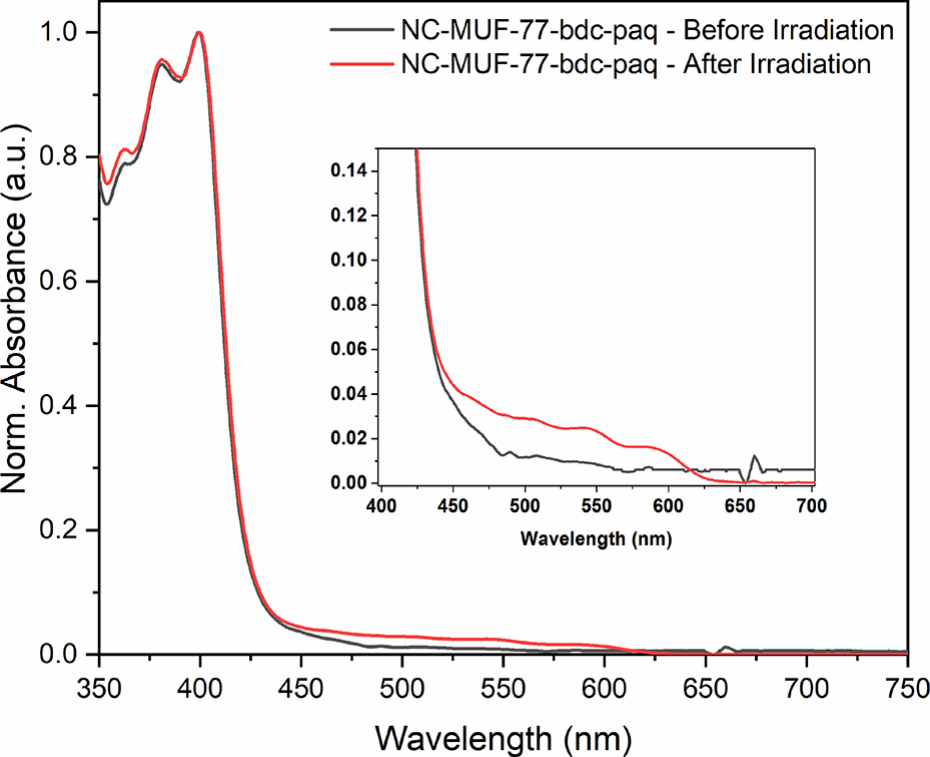
UV–visible spectra showing changes upon irradiation for NC-MUF-77-bdc-paq.

We collected the UV–visible spectra on suspensions of NC-MUF-7 and NC-MUF-77 in DMF using a CloudSpec spectrometer, which is adapted to measure the spectra of cloudy solutions. For NC-MUF-77-bdc-paq, absorption bands were seen below 450 nm, corresponding to the yellow color of the sample (Figure 4). Upon irradiation, a new absorption feature was observed in the 450–625 nm region, which corresponds to the yellow-to-red photochromic color change. Similar behavior was seen for the other photochromic frameworks with new features appearing in the visible wavelength region after irradiation (Figures S19 and S20). The intensities of the new peaks were relatively low. This can be explained on the basis that the incident light of the spectrometer simultaneously reverses the photochromism. Another factor that comes into play is the efficiency of the process causing photochromism, which may be low, contributing to the weak intensities of the new peaks.

We also collected the emission spectra of NC-MUF-7-bdc-dpq in DMF (λ_ex_ = 390 nm, Figure S21). The emission peaks were centered around 500 nm, indicating a blue-green emission. Upon irradiating this sample, the peak intensity dropped significantly, indicating the formation of a paramagnetic species upon irradiation. NC-MUF-7-bdc-dpq-OMe showed no emission peaks when suspended in DMF, but a strong emission was centered at 488 nm when suspended in dichloromethane (Figure S22). NC-MUF-77-bdc-thenil showed only a slight change of emission spectra before and after irradiation (Figure S23). These experiments show that the process responsible for photochromism in NC-MUF-77-bdc-dpq-OMe is very efficient when compared to NC-MUF-7-bdc-dpq and NC-MUF-77-bdc-thenil.

The photochromism is quenched by dissolved oxygen and inhibited by the TEMPO radical ((2,2,6,6-tetramethylpiperidin-1-yl)oxyl).^32^ For TEMPO, this was demonstrated by treating the nanocrystalline samples with a solution of TEMPO in DMF (6.5 mM) and then exposing them to sunlight. No color change was detectable, showing that TEMPO suppresses the MOF photochromism (Figure S7). To test the effect of dissolved oxygen, we bubbled an irradiated, red-colored NC-MUF-77-bdc-paq sample with oxygen gas. This immediately reversed the color change with the crystals reverting to a yellow hue (Figure S8). This quenching and inhibition of the photochromism by paramagnetic additives indicate that the photochromism in these MOF materials relies on radical formation.^33^

Polar, aprotic solvents stabilize the photogenerated radicals in these materials. After irradiating single crystals of MUF-77-bdc-paq with 405 nm light, we found that the red coloration is sustained for longer than 25 min when the crystals are immersed in DMF or *N*,*N*-dibutylformamide (DBF) (Figure S9). In dimethylsulfoxide (DMSO) , the color is sustained for only a few minutes. Immersion of the crystals in solvents of low polarity, such as dichloromethane, chloroform, and hexane, suppressed the photochromism entirely. These observations show that photoinduced proton transfer is unlikely to be the cause of the photochromism.^34,35^

Both the hmtt and bdc ligands are required in the framework to observe photochromism. We deduced this by making an IRMOF-1 (MOF-5) analogue using H_2_bdc-paq and zinc acetate (NC-IRMOF-1-bdc-paq; Scheme S6). This material has the formula [Zn_4_O(bdc-paq)_3_], and its PXRD pattern matches that of IRMOF-1 (Figure S6).^36^ When IRMOF-1-bdc-paq nanocrystals were irradiated, no photochromism was observed since no hmtt ligand is present.

A solid-state mixture of H_3_hmtt and H_2_bdc-dpq is not photochromic (Figure S10). On the other hand, a DMF or DBF solution of H_3_hmtt and H_2_bdc-dpq is photochromic (Figures S11 and S12). The photochromism is suppressed by either adding TEMPO or changing the solvent to acetonitrile. These experiments indicate that DMF or DBF can act as electron donors to partner H_2_bdc-quin ligands in electron transfer processes.

### Electron Paramagnetic Resonance Spectroscopy

**Figure 5:**
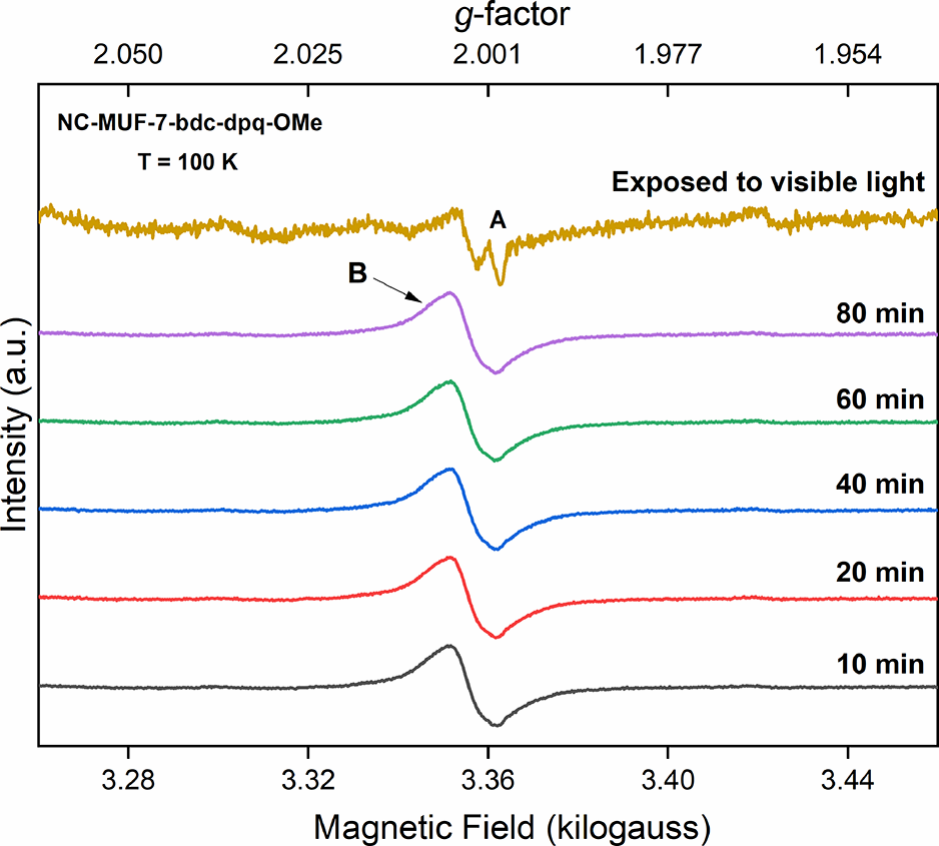
EPR spectra of NC-MUF-7-bdc-dpq-OMe as a DMF suspension at 100 K after irradiation with 405 nm light. Signal **A** arises from the quartz sample tube. Signal **B** (*g* = 2.0037) arises after irradiation, and its intensity does not change when the sample is kept in the dark inside the spectrometer cavity for up to 80 min. The signal disappears after deliberately exposing the sample to visible light (yellow spectrum).

EPR spectroscopy strongly supported our hypothesis that radicals are generated by irradiating these photochromic MOFs. We measured the EPR spectra of frozen DMF suspensions of nanocrystalline forms of the materials listed in Table 1. We first obtained an EPR spectrum on the framework suspension prior to irradiation at 100 K, and then the sample was removed from the spectrometer, placed in the dark, warmed to room temperature, and illuminated with a 405 nm laser pointer to induce the photochromism. The irradiated sample obtained in this way was immediately recooled to 100 K, and a series of EPR spectra were recorded over time (Figure 5 and Figures S13–S18). Finally, the sample was warmed again and exposed to visible light and a third spectrum was collected at 100 K.

An EPR signal with *g* = 2.0037 (signal **B**) was observed after irradiating the samples (Figure 4 and Figures S10–S14) with varying intensities. An additional very weak signal with constant intensity is also observed prior to irradiation which originates from the quartz sample tube.^37^ Signal **B** is characteristic of an organic radical, presumably a pyrazine radical anion based on its *g* factor,^38−40^ and its appearance is consistent with the photochromism correlating with radical formation. The intensity of this signal was maintained when the sample was kept in the dark environment inside the spectrometer cavity. As expected, the signal disappeared after the sample was warmed to room temperature and exposed to visible light as shown for NC-MUF-7-bdc-dpq-OMe (Figure 5). Similar EPR signals were observed for most other MOF materials (Figures S13–S15). An exception was noted for MUF-77-bdc-thenil, which exhibited an extremely weak EPR signal after irradiation (Figure S17). This is consistent with its barely perceptible color change on sunlight exposure, indicating that the bdc-thenil unit resists radical formation.

### X-ray Crystallography

**Figure 6:**
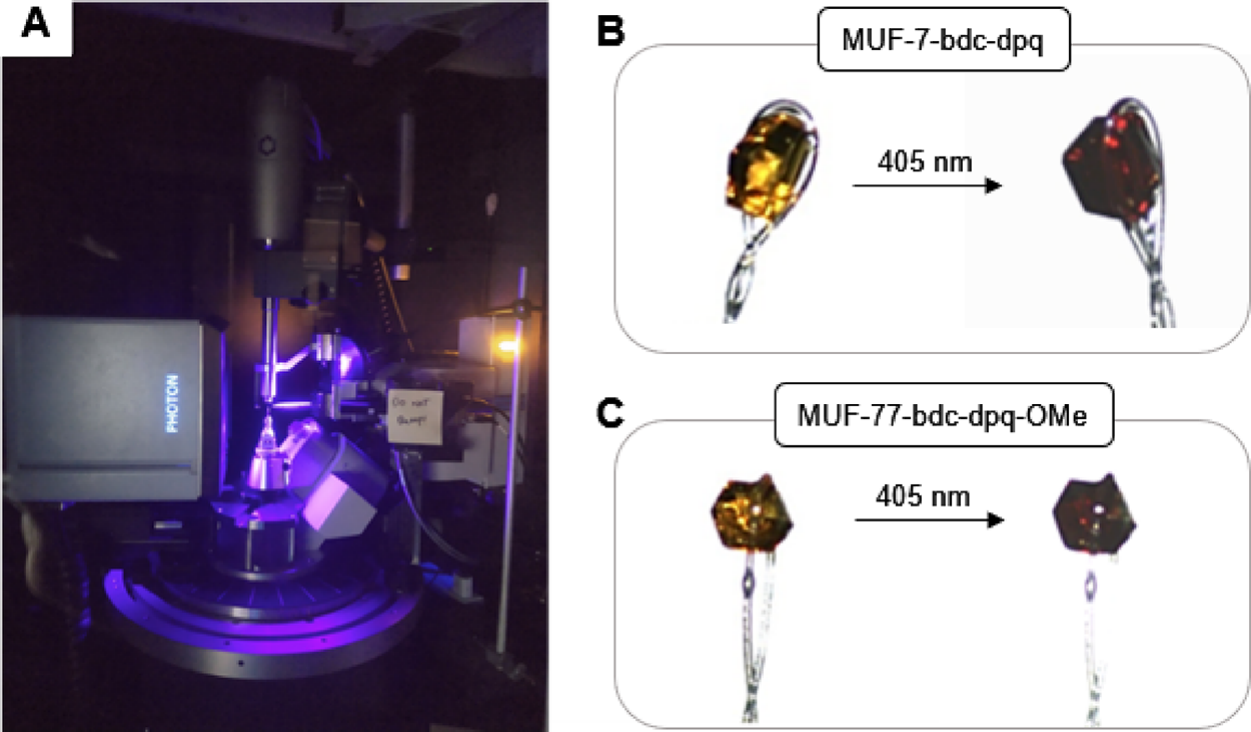
(A) Irradiation of single crystals mounted on the X-ray diffractometer with 400 nm light during data collection. (B) Photographs of a MUF-7-bdc-dpq crystal before and after irradiation with 405 nm light. (C) Photographs of a MUF-7-bdc-dpq-OMe crystal before and after irradiation with 405 nm light.

We then aimed to decipher the structure of the radical and probe the changes in bond lengths upon irradiation using SCXRD. We obtained crystal structures of MUF-7-bdc-dpq and MUF-7-bdc-dpq-OMe (i) prior to irradiation and (ii) while being illuminated with a 405 nm UV lamp (Figure 6A and Tables S3 and S4). The crystals were bathed in a stream of dry nitrogen during data collection to avoid radical quenching by atmospheric oxygen. The color change to the crystals upon irradiation was distinct (Figure 6B,C) and contrasted with the parent MUF-77 framework, [Zn_4_O(hmtt)_4/3_(bpdc)_1/2_(bdc)_1/2_], whose appearance is unchanged by UV irradiation.^27^

Upon irradiation, MUF-7-bdc-dpq showed a unit cell length decrease from 59.5745 to 59.485 Å. We focused on the bdc-dpq ligand in this framework (Table S5). The C_2_–C_3_ bond lengthens by 0.031 Å (2.2%) from 1.365 to 1.396 Å (Figure S24c). The C–C bonds of the pendant phenyl rings also show significant changes, ranging from a maximum shortening of 0.039 Å (2.6%) for the C_6_–C_7_ bond to 0.024 Å (1.7%) for the C_11_–C_12_ bond. The C–N bond lengths remained unchanged. In the hmtt ligand, the maximum length change is a shortening of 0.07 Å for the C_27_–C_28_ bond and C_46_–C_47_ bonds, which are the bonds connecting the aromatic rings to the carboxylate groups (Figure S24a,b). The bonds of the central phenyl rings show contractions in length upon irradiation, namely, C_21_–C_32_, C_32_–C_33_, C_51_–C_52_, and C_63_–C_64_ bonds, which show decreases of 0.03, 0.053, 0.036, and 0.026 Å, respectively. The aliphatic bonds, i.e., C_21_–C_22_ and C_52_–C_53_, show increases of 0.04 and 0.05 Å, respectively.

The SCXRD structures of MUF-7-bdc-dpq-OMe with and without UV irradiation showed even greater bond length changes upon irradiation than MUF-7-bdc-dpq, consistent with the larger decrease in the unit cell length (59.8236 to 59.551 Å, Table S4). The C_3_–C_3_ bond of the bdc-dpq-OMe ligand contracted by 0.05 Å (Table S6). The N_5_–C_6_ bond of the pyrazine ring lengthens by 0.057 Å (4.2%), and the C_6_–C_6_ bond contracts from 1.44 to 1.38 Å. The bond length changes extend to the very periphery of the ligand, with the C_10_–O_13_ bond lengthening by 0.033 Å, meaning that the methoxy group aids in the stabilization of the photogenerated radical. Bond length changes were also seen in the hmtt ligand, which ranged from 0.044 to 0.084 Å. The C_20_–C_21_ bond of the central phenyl ring lengthens by 0.06 Å (4.2%), while the C_66_–C_67_ bond belonging to the C_3_–symmetric hmtt ligand showed a similar lengthening of 0.071 Å (5.0%). The bonds constituting the central phenyl ring show length contractions of 0.078 Å (C_21_–C_32_) and 0.053 Å (C_51_–C_52_), while the C_20_–C_21_ bond expands by 0.06 Å.

A series of important conclusions can be drawn from this SCXRD investigation: (i) Distinctive bond length changes within the bdc-dpq and bdc-dpq-OMe ligands occurred upon irradiation. (ii) The bonds in the hmtt ligand also showed variations in length, which meant that they were also involved in the photochromism. (iii) While we cannot be certain which fraction of the ligands in these MOFs undergo a change, the bond length modifications in these MOFs are consistent with those reported for organic pyrene-fused azaacene and indeno[2,1-*a*]fluorene-11,12-dione radical anions and *N*,*N′*-diphenyldihydrophenazine radical cations.^41−43^

**Figure 7:**
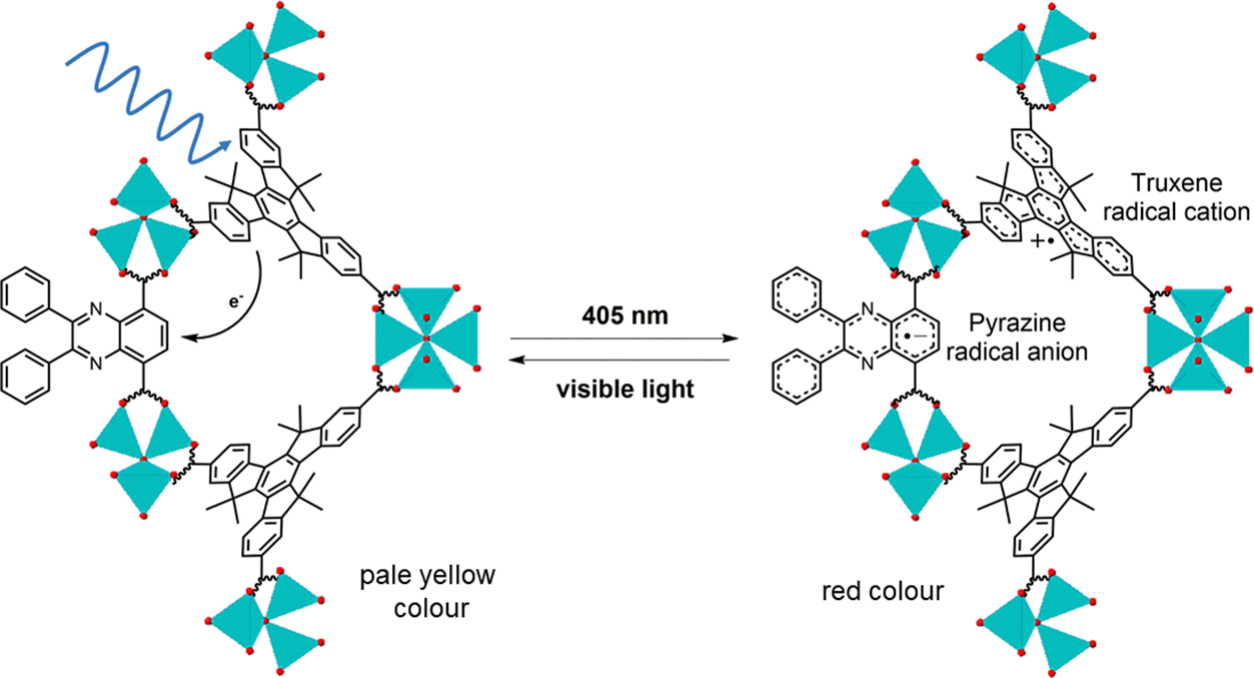
Photochromism of multicomponent MOFs with quinoxaline units. Electron transfer occurs from the truxene-based ligand to the quinoxaline group appended to the bdc ligand. The MUF-7-bdc-dpq framework is presented as an example here. Zn(II) ions are shown as turquoise tetrahedra, and oxygen atoms are shown as red spheres.

These observations indicate that a ligand-to-ligand charge transfer process takes place between the truxene and quinoxaline groups to generate a pair of radicals and render the MOFs photochromic (Figure 7). The framework structure enables electron transfer by placing the truxene and quinoxaline moieties in close proximity. The minimum distances between the truxene and quinoxaline ligands are around 3.6 Å, thus favorable for electron transfer.^44^ Truxenes are electron-rich and truxene derivatives have been used as electron donors in semiconductor devices.^45−47^ In a complementary way, quinoxalines are well-known electron acceptors.^48^ Irradiation of these frameworks at 405 nm thus induces electron transfer from the truxene-based hmtt ligands to the bdc-quin ligands to generate a truxene radical cation and a pyrazine radical anion. Similar electron transfer processes have been observed in fluorene-quinoxaline push-pull conjugated polymers.^49,50^

## Conclusions

In the multicomponent MOFs investigated here, the truxene ligand can be viewed as a cyclic trimer of fluorene. The architecture of the MUF-7 and MUF-77 frameworks aids in the longevity of these radicals. The truxene and bdc ligands are sufficiently close that through-space electron transfer can take place, while once delocalized, the radicals are sufficiently isolated from one another to inhibit quenching by simple recombination. Additionally, the electron-donating ability of the methoxy group in bdc-dpq-OMe means that the radical is more effectively delocalized in MUF-7-bdc-dpq-OMe by the captodative effect.^35,51^ In contrast, photochromism is quenched by the installation of thenil substituents on the quinoxaline unit. The emission spectra also confirm these observations as the fluorescence intensities are quenched upon irradiation in DMF for MUF-7-bdc-dpq and MUF-7-bdc-dpq-OMe but not for MUF-77-bdc-thenil.

Taken together, these results underscore the utility of the multicomponent approach to the design and construction of photochromic MOFs. Complementary electron donor and acceptor units can be installed on the framework components and then positioned in fixed, predetermined locations to allow photochromism by through-space electron transfer. The stability of the radical and, thus, the photochromic properties can be tuned via functionalization of the component ligands.

## Supporting Information Available

Crystal structure data for MUF-7-bdc-dpq before irradiation (CIF), MUF-7-bdc-dpq with irradiation (CIF), MUF-7-bdc-dpq-OMe before irradiation (CIF), and MUF-7-bdc-dpq-OMe with irradiation (PDF)
Crystal structure data for MUF-7-bdc-dpq before irradiation (CIF), MUF-7-bdc-dpq with irradiation (CIF), MUF-7-bdc-dpq-OMe before irradiation (CIF), and MUF-7-bdc-dpq-OMe with irradiation (CIF)

## Supplementary Material

cm2c02220_si_001.pdf

cm2c02220_si_002.cif

cm2c02220_si_003.cif

cm2c02220_si_004.cif

cm2c02220_si_005.cif

## Notes

Crystal structures are deposited at ccdc.cam.ac.uk with deposition numbers 2189834, 2189835, 2189536, 2189833, and 2191826.
