# Supplementary material for: Photoinduced Electron Transfer in Multicomponent Truxene-Quinoxaline Metal–Organic Frameworks
Source: Chem Mater. 2022 Sep 15;34(18):8437–45. doi: 10.1021/acs.chemmater.2c02220 (PMC10242685; doi:10.1021/acs.chemmater.2c02220)
Supplement: Supplementary file 1 — cm2c02220_si_001.pdf [file cm2c02220_si_001.pdf]

Electronic Supporting Information for

Photoinduced Electron Transfer in Multicomponent Truxene-Quinoxaline Metal-Organic Frameworks

Joel Cornelio,[a] Seok June Lee,[a] Tian-You Zhou,[a] Adil Alkas,[a] Kavipriya Thangavel,[b] Andreas Pöppl,[b] and Shane G. Telfer[a]*

*[a] School of Natural Sciences, MacDiarmid Institute of Advanced Materials and Nanotechnology, Massey University, Palmerston North 4410, New Zealand. *E-mail: s.telfer@massey.ac.nz*

[b] Felix Bloch Institute for Solid State Physics, Leipzig University, Linnestrasse 5, D-04103, Leipzig, Germany.

Contents

1. General Information 2
2. Ligand Synthesis and Characterisation 2
3. General procedure A for the synthesis of quinoxaline esters 3
4. General procedure B for the synthesis of H_2bdc-quin ligands 5
5. Synthesis of photochromic MUF-7 and MUF-77 single crystals 7
6. Synthesis of nanocrystalline, photochromic MUF-7 and MUF-77 8
7. PXRD of photochromic MOFs 10
8. Synthesis and PXRD of IRMOF-1-bdc-paq 12
9. Exposure of nanocrystalline (NC) MOFs to sunlight 13
10. Bubbling of oxygen gas 13
11. Effect of solvent on photochromism 14
12. Exposure of solid state mixtures and solutions to irradiation 15
13. Electron Paramagnetic Resonance Spectroscopy 16
14. UV-Visible Spectra of NC-MUF-7 and NC-MUF-77 suspensions 21
15. Emission Spectra 23
16. Crystallographic details and bond length analysis 25
17. ^{1}H NMR spectra of digested MOFs 32
18. References 43

1. General Information

All starting materials and solvents were used as received from commercial sources without further purification unless otherwise noted. NMR spectra were collected at room temperature using a Bruker Avance 400 MHz or 500 MHz spectrometer, with the use of the solvent proton as an internal standard. Emission spectra were recorded with a Horiba Scientific Fluoromax-4 Spectrofluorimeter. Spectra were corrected for detector and grating efficiencies using FluorEssence, the in-house software of the fluorimeter. X-ray diffraction data were collected using a Bruker D8 Venture diffractometer with Cu_{α} radiation (wavelength = 1.54180 Å), with a diamond microfocus X-ray source and a Photon III 28 detector. For PXRD, the collected data were processed using APEX3. For SCXRD, the crystals were exchanged with *N*,*N*-dibutylformamide (DBF) and mounted on a nylon loop using paratone oil. The crystal was kept under an atmosphere of nitrogen at 293 K for all measurements.

EPR spectra were measured using a Bruker EMX Micro X-band spectrometer with a microwave power of 0.2 mW. Samples were homogenised using an ultrasonic bath and inserted into a Helium Oxford Cryostat ESR900 and spectra were recorded at 100 K. Samples were removed and warmed to room temperature before illumination with a 405 nm laser. The illuminated samples were cooled to 100 K and EPR spectra were recorded. Finally, samples were warmed again, exposed to visible light until the colour returned to yellow. After cooling to 100 K and another EPR spectrum was collected.

2. Ligand Synthesis and Characterisation

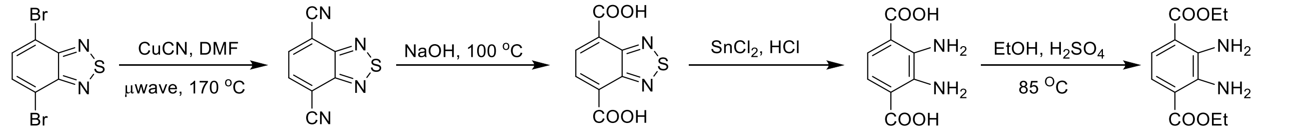

1 2 3 4 5

Scheme S1: Synthesis of **5**.

Compound 2

A total of five 1.25 g portions of **1** (6.25 g, 21.2 mmol, 1 eq.) were mixed with five 1.25-gram portions of CuCN (6.25 g, 70.1 mmol, 3.3 eq.) in five microwave reaction tubes. Dry DMF (5 mL) was added to each tube. The tubes were heated in a microwave reactor at 170 °C for 2 hours each. The portions were combined, acidified $FeCl_3$ solution was added and stirred for 30 minutes. The mixture was extracted with dichloromethane. The organic layers were collected, the solvent dried *in vacuo* and yield the product. **Yield:** 2.6 g, 13.8 mmol, 65%.^{1}H NMR (500 MHz, $CDCl_3$) δ 8.16 (s, 2H).[1]

Compound 3

Compound **2** (2.6 g, 13.8 mmol) was dissolved in 80 mL of 20% aq. NaOH solution and heated at 100 °C for 3 hours. The flask was transferred to an ice bath and neutralised with conc. HCl to a pH of 2 to precipitate a brown solid. The solid was washed with water, filtered and dried. **Yield:** 1.6 g, 7.1 mmol, 51%. ^{1}H NMR (500 MHz, $CDCl_3$) δ 8.22 (s, 2H).[1]

Compound 4

A mixture of compound **3 (**1.5 g, 6.7 mmol) $SnCl_2$ (7.2 g, 38 mmol) and conc. HCl (20 mL) was stirred overnight at room temperature. The solid obtained was filtered and washed with water and dried to give the product.

Yield: 0.97 g, 4.94 mmol, 74%. ^{1}H NMR (500 MHz, $CDCl_3$) δ 7.01 (s, 2H). ^{13}C NMR (125 MHz, DMSO-d_6) 170.41, 140.67, 117.07, 111.78. HRMS (ESI) m/z: $[m-H]^-$ calc. for $C_8H_7N_2O_4$: 195.0411, Found: 195.0408.

Compound 5

Compound **4** (0.85 g, 4.3 mmol) was suspended in 70 mL of EtOH, and conc. H_2SO_4 (7 mL) was added dropwise. The reddish-brown solution was heated at 85 °C for 48 hours. The EtOH was evaporated *in vacuo.* The residual reaction mixture was added dropwise into an ice-cold suspension of aqueous saturated K_2CO_3 to give a brown solid. The solid obtained was extracted with DCM and water. The collected organic layers were evaporated *in vacuo* to yield the product. **Yield:** 0.65 g, 2.58 mmol, 59%. ^{1}H NMR (500 MHz, $CDCl_3$) δ 7.32 (s, 2H), 4.35 (q, 4H), 1.40 (t, 6H). ^{13}C NMR (100 MHz, $CDCl_3$) δ 168.22, 140.83, 118.29, 114.41, 60.76, 14.28. HRMS (ESI) m/z: $[m+H]^+$ calc. for $C_{12}H_{17}N_2O_4$: 253.1182, Found: 253.1180.

3. General procedure A for the synthesis of quinoxaline esters

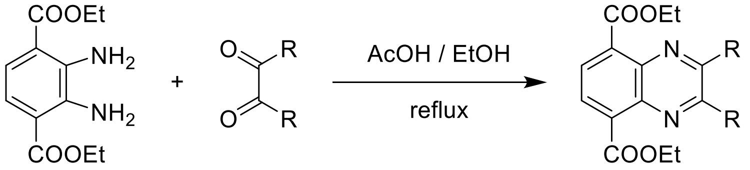

5 **6, 7, 8, 9, 10**

Scheme S2: Synthesis of bdc-quin esters.

In a typical synthesis, compound **5** (1 eq.) was combined with the diketone (1.1 eq.). 9 mL of 1:2 EtOH/AcOH was added, and the mixture was heated on reflux for 3 hours. Anhydrous $MgSO_4$ was added, and the refluxing was continued for an additional 12 hours. The reaction mixture was added to water dropwise to precipitate out the crude product, which was filtered. The residue was washed with 1:1 hexane:DCM (this fraction was rejected) followed by a wash with pure DCM and MeOH which was collected and dried to give compounds **6**, **7**, **8**, **9**, and **10**.

**6** 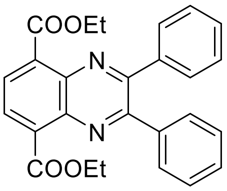	Made by reacting compound **5** (100 mg, 0.396 mmol, 1 eq.) and benzil (91 mg, 0.436 mmol, 1.1 eq.) acc. to general procedure A. **Yield:** 135 mg, 0.316 mmol, 79%. ^{1}H NMR (500 MHz, $CDCl_3$, ppm) δ 8.14 (s, 2H, H_a), 7.70 (d, *J* = 7.3 Hz, 2H), 7.44 – 7.34 (m, 6H), 4.57 (q, *J* = 7.1 Hz, 2H), 1.52 (t, *J* = 7.1 Hz, 3H). ^{13}C NMR (100 MHz, $CDCl_3$) 166.36, 152.99, 138.37, 133.95, 129.77, 129.50, 128.28, 91.90, 14.41. HRMS (ESI) m/z: $[m+H]^+$ calc. for $C_{26}H_{23}N_2O_4$: 427.1613, Found: 427.1646.

**7**	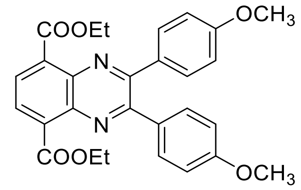	Made by reacting compound **5** (100 mg, 0.396 mmol, 1 eq.) and 4,4'-dimethoxybenzil (128 mg, 0.436 mmol, 1.1 eq.) acc. to general procedure A. **Yield:** 158 mg, 0.324 mmol, 82 %. ^{1}H NMR (500 MHz, $CDCl_3$) δ 8.08 (s, 2H), 7.70 (d, *J* = 8.3 Hz, 2H), 6.90 (d, *J* = 8.4 Hz, 2H), 4.57 (q, *J* = 7.2 Hz, 4H), 3.86 (s, 6H), 1.53 (t, *J* = 7.1 Hz, 6H). ^{13}C NMR (100 MHz, $CDCl_3$) δ 166.46, 160.77, 152.32, 138.06, 133.59, 131.60, 130.97, 129.33, 113.77, 61.79, 55.32, 14.43. HRMS (ESI) m/z: $[m+H]^+$ calc. for $C_{28}H_{27}N_2O_6$: 487.1864, Found: 487.1856
**8**	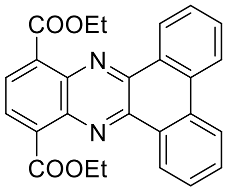	Made by reacting compound **5** (100 mg, 0.396 mmol, 1 eq.) and 9,10-phenanthrenequinone (90 mg, 0.436 mmol, 1.1 eq.) acc. to general procedure A. **Yield:** 160 mg, 0.376 mmol, 95%. ^{1}H NMR (500 MHz, $CDCl_3$) δ 9.39 (d, *J* = 8.0 Hz, 2H), 8.60 (d, *J* = 8.0 Hz, 2H), 8.24 (s, 2H), 7.85 (t, *J* = 15.2, 7.2 Hz, 2H), 7.78 (t, *J* = 7.5 Hz, 2H), 4.69 (q, *J* = 7.1 Hz, 4H), 1.64 – 1.60 (t, 6H). ^{13}C NMR (125 MHz, $CDCl_3$) δ 166.68, 142.76, 139.29, 134.47, 132.47, 130.96, 129.86, 129.55, 128.08, 126.78, 123.04, 61.90, 14.48. HRMS (ESI) m/z: $[m+H]^+$ calc. for $C_{26}H_{21}N_2O_4$: 425.1423, Found: 425.1491.
**9**	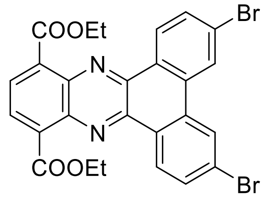	Made by reacting compound **5** (100 mg, 0.396 mmol, 1 eq.) and 2,6-dibromophenanthrene-9,10-dione (160 mg, 0.436 mmol, 1.1 eq.)[2] acc. to general procedure A. **Yield:** 170 mg, 0.291 mmol, 74%. ^{1}H ^{1}H NMR (500 MHz, $CDCl_3$, ppm) ^{1}H NMR (500 MHz, $CDCl_3$) δ 9.17 (d, *J* = 8.5 Hz, 2H), 8.59 (s, 2H), 8.26 (s, 2H), 7.88 (d, *J* = 8.5 Hz, 2H), 4.67 (q, *J* = 14.2, 7.1 Hz, 4H), 1.60 (t, *J* = 7.1 Hz, 6H).^{13}C NMR (100 MHz, $CDCl_3$) δ 166.31, 141.61, 139.32, 134.35, 132.48, 131.83, 130.21, 128.81, 128.39, 126.23, 126.00, 61.95, 14.59. HRMS (ESI) m/z: $[m+H]^+$ calc. for $C_{26}H_{18}Br_2N_2O_4$: 582.9633, Found: 582.9693.
**10**	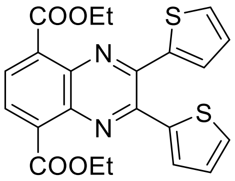	Made by reacting compound **5** (100 mg, 0.396 mmol, 1 eq.) and 2,2'-thenil (97 mg, 0.436 mmol, 1.1 eq.) acc. to general procedure A. **Yield:** 120 mg, 0.273 mmol, 69%. ^{1}H NMR (500 MHz, $CDCl_3$, ppm) δ 8.09 (s, 2H), 7.56 (d, *J* = 5.0 Hz, 2H), 7.51 (d, *J* = 3.7 Hz, 2H), 7.06 (t, 2H), 4.58 (q, *J* = 14.2, 7.1 Hz, 4H), 1.55 (t, *J* = 7.1 Hz, 6H). ^{13}C NMR (100 MHz, $CDCl_3$) δ 170.90, 150.90, 145.99, 142.01, 138.47, 135.86, 135.44, 134.39, 132.65, 66.57, 19.30. HRMS (ESI) m/z: $[m+H]^+$ calc. for $C_{22}H_{19}N_2O_4S_2$: 439.0781, Found: 439.0781.

4. General procedure B for the synthesis of H_2bdc-quin ligands

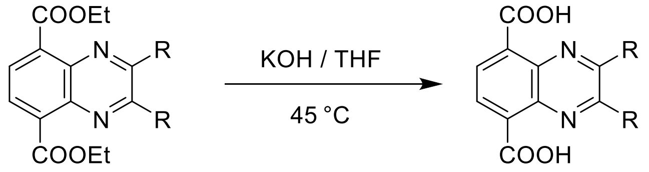

6, 7, 8, 9, 10 **H_2bdc-quin**

Scheme S3: Synthesis of bdc-quin ligands.

For obtaining quinoxaline carboxylic acid ligands by hydrolysis, the diethylesters (**6**, **7**, **8**, **9**, or **10**) were dissolved in 20 mL of 1:1 THF:2M aq. KOH and heated at 45 °C, overnight. The THF was removed using a separating funnel and the aqueous layer was neutralised using 2M HCl to a pH of 3-4 to precipitate out the carboxylic acids. The solid obtained was filtered and washed with water and dried to give ligands H_2bdc-dpq, H_2bdc-dpq-OMe, H_2bdc-paq, H_2bdc-paq-Br, and H_2bdc-thenil.

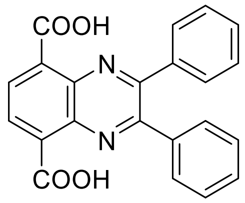

H_2bdc-dpq

Prepared by the reaction of **6** according to general procedure B. (**Yield:** 77 mg, 0.207 mmol, 84%). ^{1}H NMR (500 MHz, DMSO-d_6, ppm) δ 14.12 (bs, 1H), 8.32 (s, 2H), 7.55 (d, *J* = 7.0 Hz, 4H), 7.51 – 7.41 (m, 6H). ^{13}C NMR (100 MHz, DMSO-d_6) δ 167.03, 153.71, 138.02, 137.75, 133.45, 131.05, 130.86, 130.31, 130.12, 128.96, 128.76. HRMS (ESI) m/z: [m-H]$^-$ calc. for $C_{22}H_{13}N_2O_4$: 369.0870, Found: 369.0881.

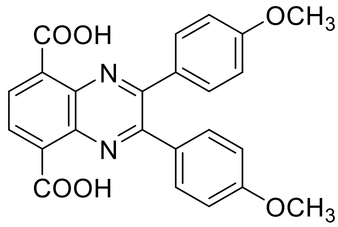

H_2bdc-dpq-OMe

Prepared by the reaction of **7** according to general procedure B. **Yield:** 110 mg, 0.255 mmol, 92%. ^{1}H NMR (500 MHz, DMSO-d_6, ppm) δ 14.26 (bs, 2H), 8.30 (s, 2H), 7.53 (d, *J* = 8.8 Hz, 4H), 7.03 (d, *J* = 8.9 Hz, 4H), 3.82 (s, 6H).^{13}C NMR (100 MHz, DMSO-d_6) δ 166.89, 161.09, 153.11, 137.51, 132.55, 131.75, 130.88, 130.15, 114.48, 55.80. HRMS (ESI) m/z: [m-H]$^-$ calc. for $C_{24}H_{17}N_2O_6$: 429.1081, Found: 429.1093.

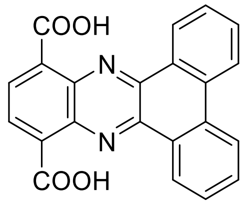

H_2bdc-paq

Prepared by the reaction of **8** according to general procedure B. **Yield:** 95 mg, 0.258 mmol, 84%. ^{1}H NMR (500 MHz, DMSO-d_6, ppm) δ 14.08 (bs, 2H), δ 9.15 (d, *J* = 7.7 Hz, 2H), 8.87 (d, *J* = 7.8 Hz, 2H), 8.39 (s, 2H), 7.98 (t, 2H), 7.91 (t, *J* = 7.3 Hz, 2H). ^{13}C NMR (100 MHz, DMSO-d_6) δ 172.18, 146.71, 143.32, 138.82, 137.25, 137.00, 135.55, 133.81, 133.55, 130.87, 129.11. HRMS (ESI) m/z: [m-H]$^-$ calc. for $C_{22}H_{11}N_2O_4$: 367.0797, Found: 367.0726.

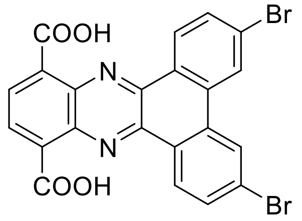<br>H_2bdc-paq-Br	Prepared by the reaction of **9** according to general procedure B. **Yield:** 120 mg, 0.273 mmol, 69%. ^{1}H NMR (500 MHz, DMSO-d_6) δ 9.03 (s, 2H, H_d), 8.85 (d, J = 8.4 Hz, 2H, H_c), 8.30 (s, 2H, H_a), 8.03 (d, J = 8.2 Hz, 2H, H_b). ^{13}C NMR (125 MHz, DMSO-d_6) δ 167.42, 141.00, 138.64, 134.66, 132.86, 132.49, 130.82, 128.20, 127.81, 127.33, 126.62. HRMS (ESI) m/z: [m-H]$^-$ calc. for $C_{22}H_8Br_2N_2O_4$: 524.8903, Found: 524.8930.
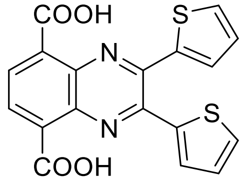<br>H_2bdc-thenil	Prepared by the reaction of **10** according to general procedure B. **Yield:** 75 mg, 0.196 mmol, 86%. ^{1}H NMR (500 MHz, DMSO-d_6) δ 13.84 (bs, 2H) 8.21 (s, 2H), 7.91 (dd, J = 5.0, 1.1 Hz, 2H), 7.41 (dd, J = 3.7, 1.1 Hz, 2H), 7.18 (dd, J = 5.0, 3.7 Hz, 2H). ^{13}C NMR (100 MHz, DMSO-d_6) δ 167.02, 146.69, 140.33, 137.10, 133.25, 131.92, 131.08, 130.79, 128.63. HRMS (ESI) m/z: [m-H]$^-$ calc. for $C_{18}H_9N_2O_4S_2$: 381.0009, Found: 380.9997.

5. Synthesis of photochromic MUF-7 and MUF-77 single crystals

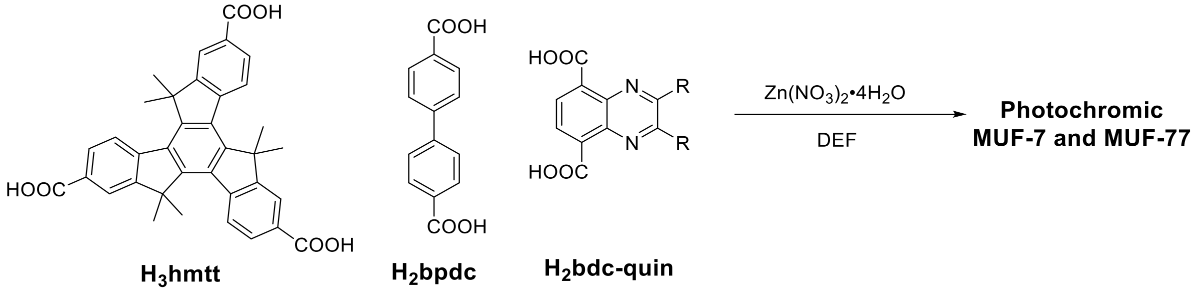

Scheme S4: Synthesis of photochromic MUF-7 and MUF-77 single crystals.

H_3hmtt, H_2bpdc, and H_2bdc-quin ligand (details in table below) were dissolved in 1 mL of anhy. *N*,*N*-diethylformamide and 50 µL of water was added. Zinc nitrate tetrahydrate was added and the sample was sonicated briefly and placed in an isothermal oven kept at 85 °C for 24 hours. The mother liquor was decanted when hot and the crystals were washed with anhydrous DMF and stored in DMF at room temperature.

Table S1: Quantities of ligands and metal salt required for synthesising photochromic MUF-7 and MUF-77 crystals.

MOF	H_3hmtt (µmol, eq.)	H_2bpdc (µmol, eq.)	H_2bdc-quin (µmol, eq.)	$Zn(NO_3)_2\cdot 4(H_2O)$ (µmol, eq.)
MUF-7-bdc-dpq	4.5 mg (8.06, 1)	2.9 mg (12.0, 1.5)	3.4 mg (9.26, 1.15)	15.8 mg (60.4, 7.5)
MUF-7-bdc-dpq-OMe	3.5 mg (6.27, 1)	1.9 mg (8.14, 1.3)	5.4 mg (12.5, 2)	12 mg (45.7, 7.3)
MUF-77-bdc-paq	4.5 mg (8.06, 1)	2.9 mg (12.0, 1.5)	3.4 mg (9.26, 1.15)	15.8 mg (60.4, 7.5)
MUF-77-bdc-paq-Br	4.5 mg (8.06, 1)	2.9 mg (12.1, 1.5)	3.5 mg (9.26, 1.15)	14.7 mg (56.3, 7.0)
MUF-77-bdc-thenil	4.5 mg (8.06, 1)	2.9 mg (12.1, 1.5)	3.5 mg (9.26, 1.15)	14.7 mg (56.3, 7.0)

6. Synthesis of nanocrystalline, photochromic MUF-7 and MUF-77

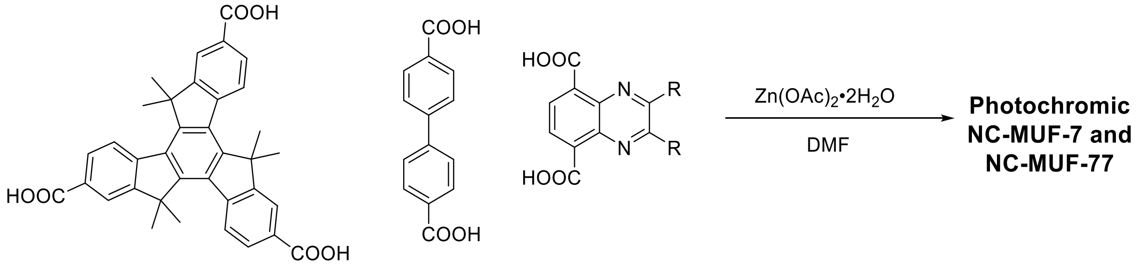

H_3hmtt **H_2bpdc** **H_2bdc-quin**

Scheme S5: Synthesis of photochromic NC-MUF-7 and NC-MUF-77 crystals.

H_3hmtt, H_2bpdc, and H_2bdc-quinoxaline ligands (details in table below) were stirred and dissolved in 2.5 mL of a 50:1 v/v solution of *N*,*N*-dimethylformamide:water. Solid $Zn(OAc)_2 \cdot 2H_2O$ was added and the stirring was continued for another 30 minutes. The resulting suspension was centrifuged thrice with fresh DMF and suspended in DMF and placed in an isothermal oven at 85 °C overnight. The crystals were centrifuged again with fresh, dry DMF and stored at room temperature.

Table S2: Quantities of ligands and metal salt required for synthesising photochromic NC-MUF-7 and NC-MUF-77 crystals.

MUF system	H_3hmtt (μmol, eq.)	H_2bpdc (μmol, eq.)	H_2quin-bdc (μmol, eq.)	$Zn(OAc)_2 \cdot 2(H_2O)$ (μmol, eq.)
NC-MUF-7-bdc-dpq	18 mg (32.2, 2.5)	3.1 mg (12.9, 1)	4.7 mg (12.9, 1)	28.3 mg (128.9, 10)
NC-MUF-7-bdc-dpq-OMe	25 mg (44.7, 2.5)	4.3 mg (17.9, 1)	7.7 mg (17.9, 1)	39.2 mg (179.0, 10)
NC-MUF-77-bdc-paq	18 mg (32.2, 2.5)	3.1 mg (12.9, 1)	4.7 mg (12.9, 1)	28.3 mg (128.9, 10)
NC-MUF-77-bdc-paq-Br	20 mg (35.8, 2.5)	3.5 mg (14.3, 1)	7.5 mg (14.3, 1)	37.2 mg (179.0, 12)
NC-MUF-77-bdc-thenil	25 mg (44.7, 2.5)	4.3 mg (17.9, 1)	6.8 mg (17.9, 1)	39.2 mg (179.0, 10)

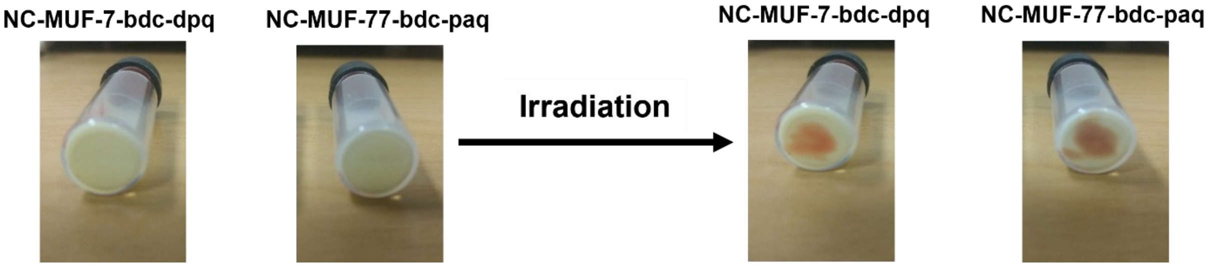

Figure S1: Photos of NC-MUF-7-bdc-dpq and NC-MUF-77-bdc-paq samples before (left) and after (right) irradiation with a 405 nm laser pointer. The red colour is seen only at the areas where the sample was irradiated.

7. PXRD of photochromic MOFs

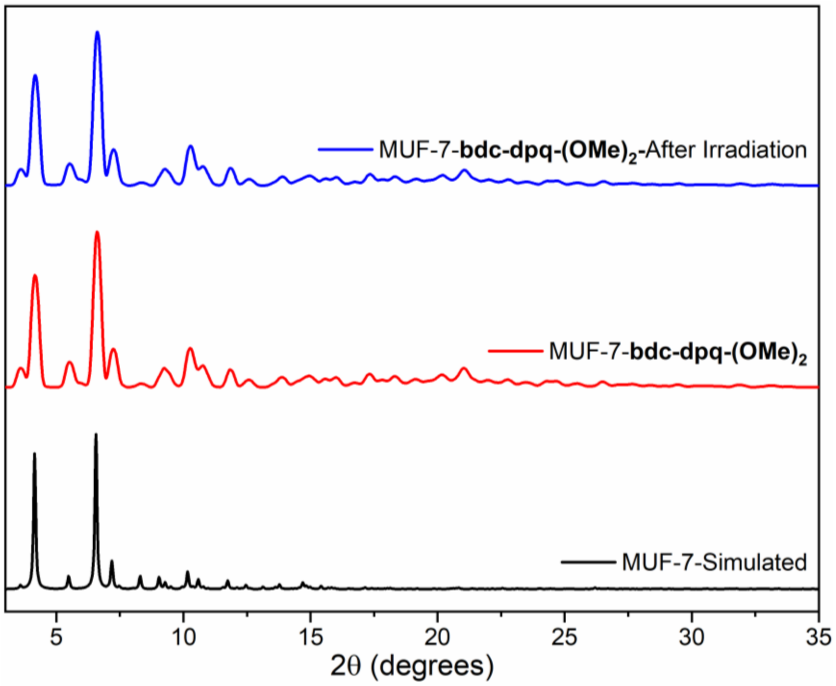

Figure S2: PXRD patterns (Cu$_\alpha$ radiation) of photochromic MUF-7-bdc-dpq-(OMe) single crystals compared to the PXRD simulated from the SCXRD structure.

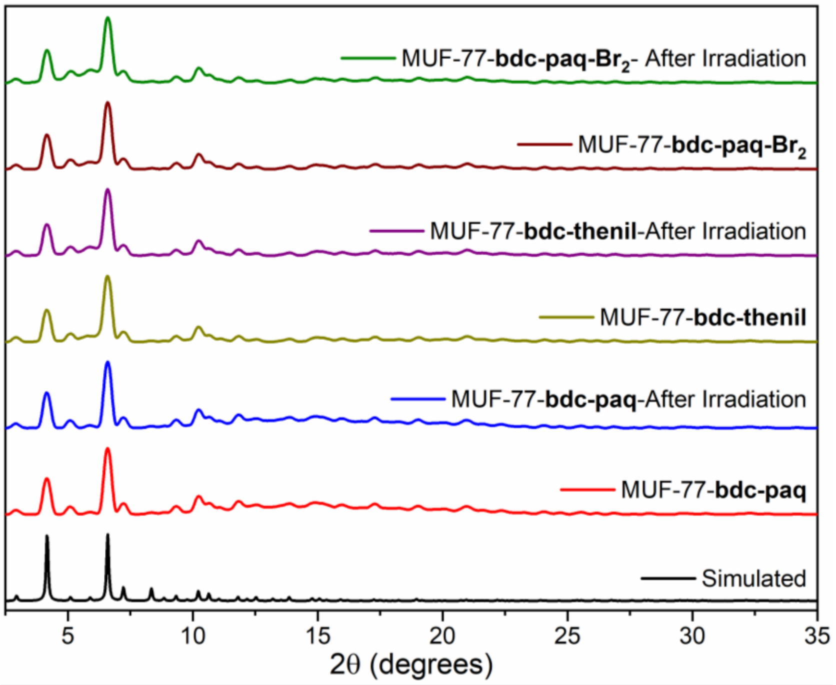

Figure S3: PXRD patterns (Cu$_\alpha$ radiation) of photochromic MUF-77 single crystals before and after UV irradiation compared to the PXRD pattern simulated from the SCXRD structure.

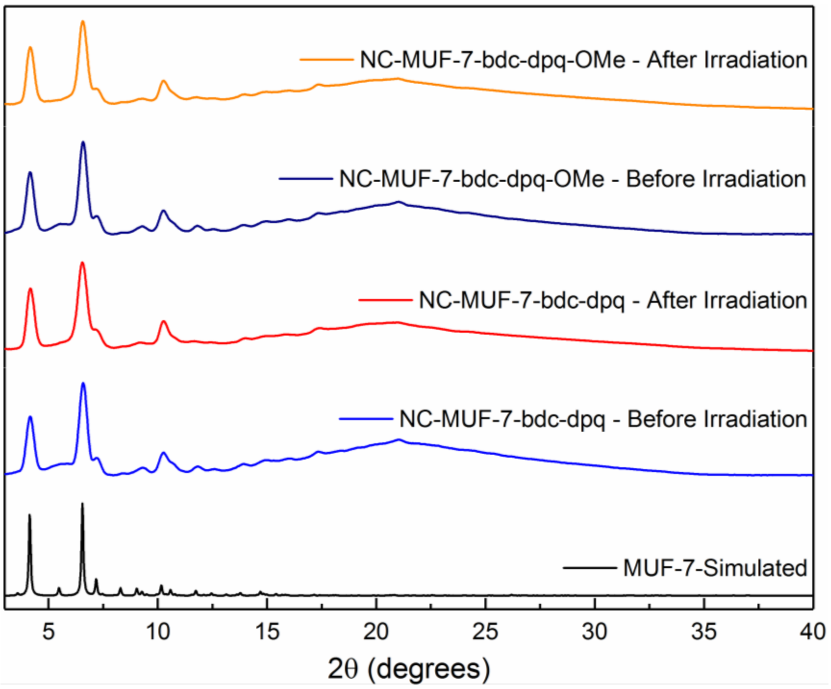

Figure S4: PXRD patterns (Cu$_\alpha$ radiation) of nanocrystalline MUF-7 crystals before and after irradiation.

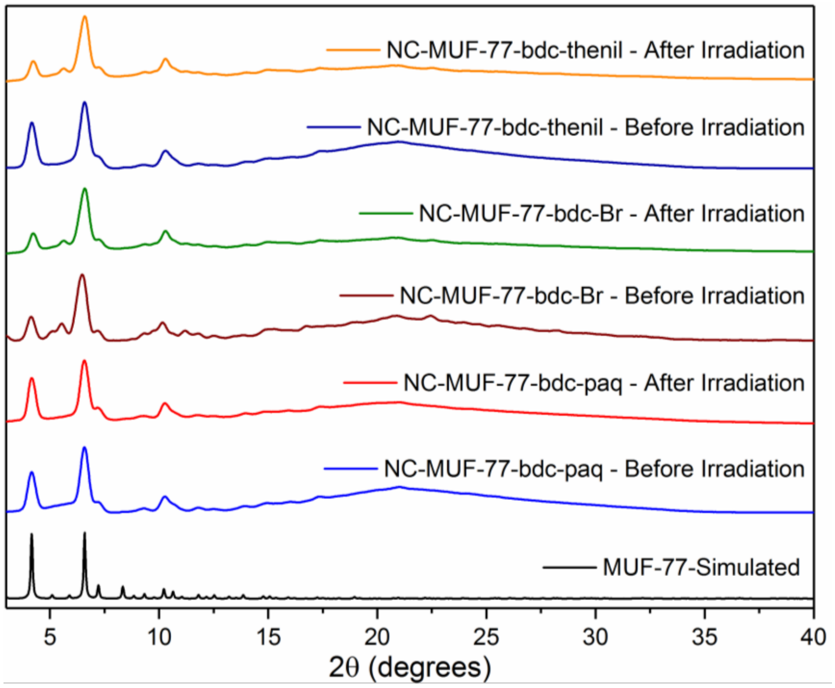

Figure S5: PXRD patterns (Cu$_\alpha$ radiation) of nanocrystalline MUF-77 crystals before and after irradiation.

8. Synthesis and PXRD of IRMOF-1-bdc-paq

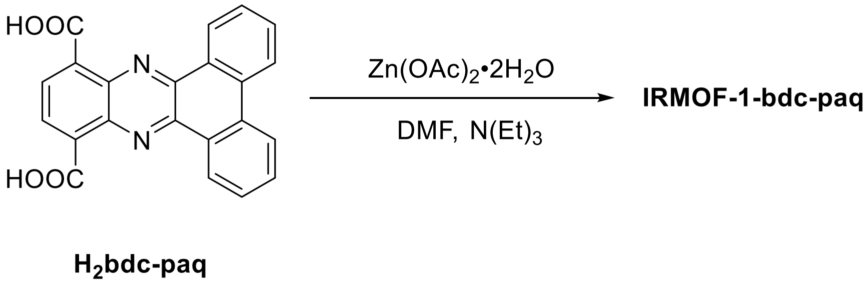

Scheme S6: Synthesis of IRMOF-1-bdc-paq.

H_2bdc-paq (10 mg, 27.1 µmol, 1eq.) and triethylamine (30 mg, 40 µl, 298 µmol) were stirred and dissolved in 2 mL of anhy. *N*,*N*-dimethylformamide. Solid $Zn(OAc)_2{\cdot}2H_2O$ (14.9 mg, 67 .8 µmol, 2.5 eq.) was added and the sample and the stirring was continued for another 30 minutes. The resulting suspension was washed and centrifuged thrice with fresh DMF.

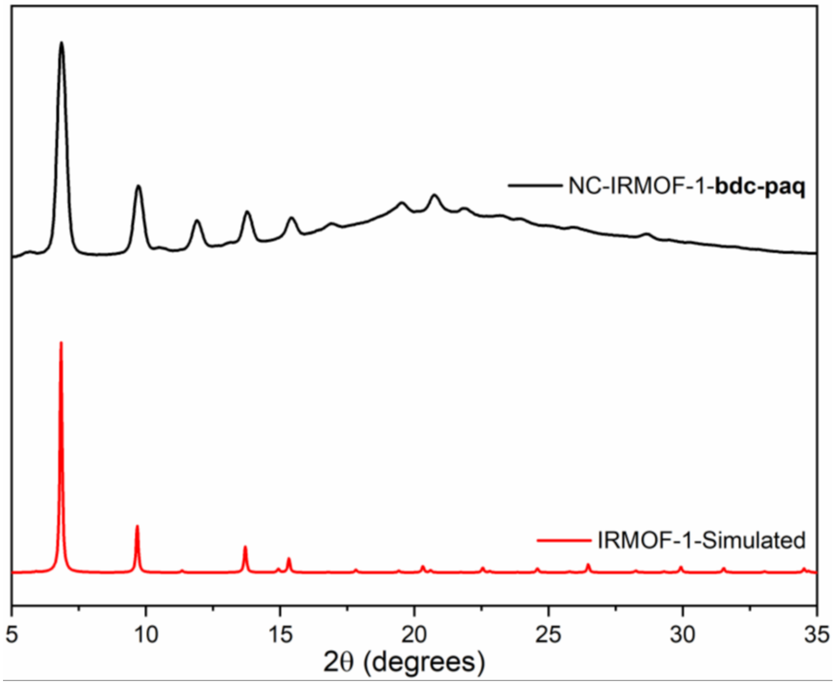

Figure S6: PXRD of nanocrystalline, IRMOF-1-bdc-paq (black) compared to PXRD pattern simulated from SCXRD structure of IRMOF-1/MOF-5 (red).[3]

9. Exposure of nanocrystalline (NC) MOFs to sunlight

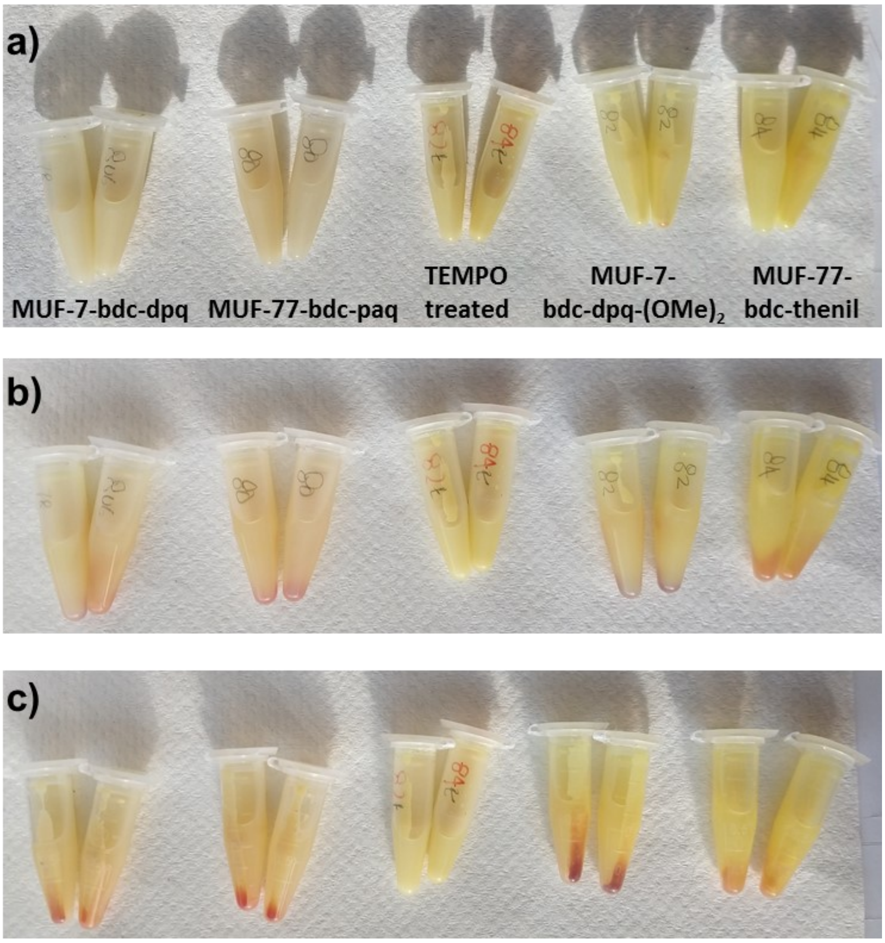

Figure S7: Photographs of **a)** NC-MUF-7 and NC-MUF-77 crystals **b)** after 5 minutes exposure to sunlight **c)** the sunlight-exposed samples with the vials turned over showing that the area more exposed to sunlight shows a stronger colour change. Note that the samples placed in the centre have been treated with TEMPO and show no photochromism.

10. Bubbling of oxygen gas

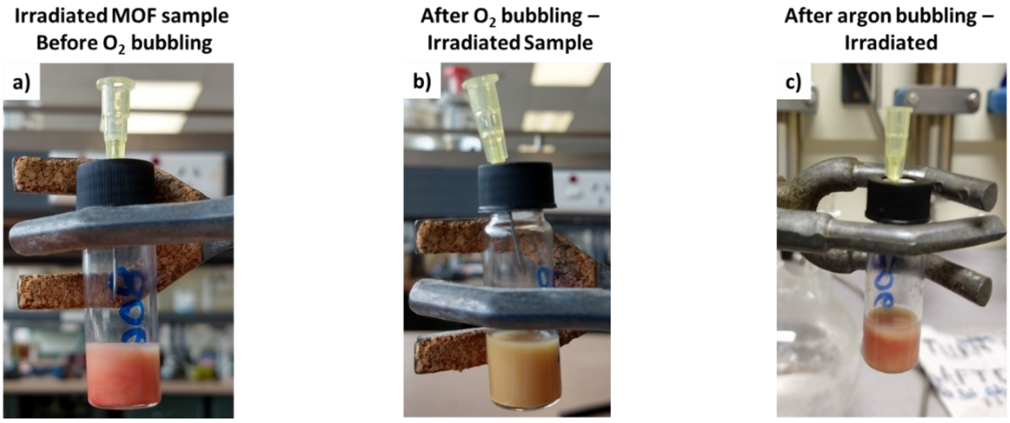

Figure S8: Photos of **a)** NC-MUF-77-bdc-paq showing photochromism after irradiation with 405 nm laser pointer. **b)** Irradiation after bubbling with oxygen gas showing no photochromism and **c)** The sample from **b)** purged with argon and then irradiated at 405 nm showing the reappearance of photochromism.

11. Effect of solvent on photochromism

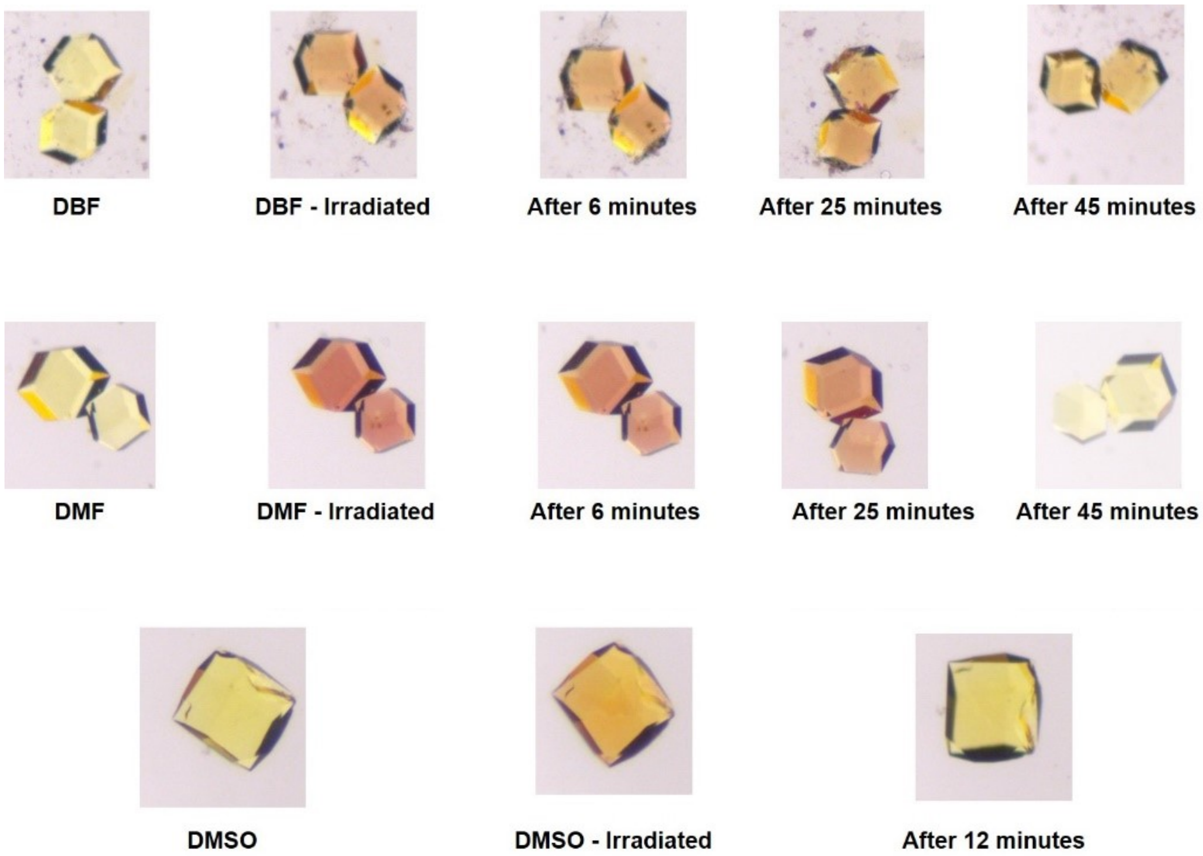

Figure S9: Crystals of MUF-77-bdc-paq suspended in *N*,*N*-dibutylformamide (DBF), *N*,*N*-dimethylformamide (DMF), and dimethylsulfoxide (DMSO) at different time intervals after irradiation with a 405 nm laser pointer for 10 seconds.

12. Exposure of solid state mixtures and solutions to irradiation

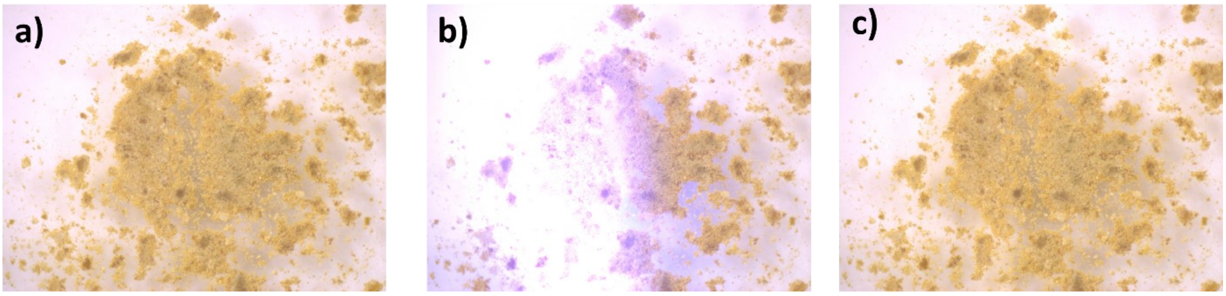

Figure S10: Effect of irradiation of 405 nm laser pointer light on a solid state mixture of H_3hmtt and H_2bdc-dpq. **a)** before irradiation, **b)** with irradiation and **c)** after irradiation. No photochromism is observed.

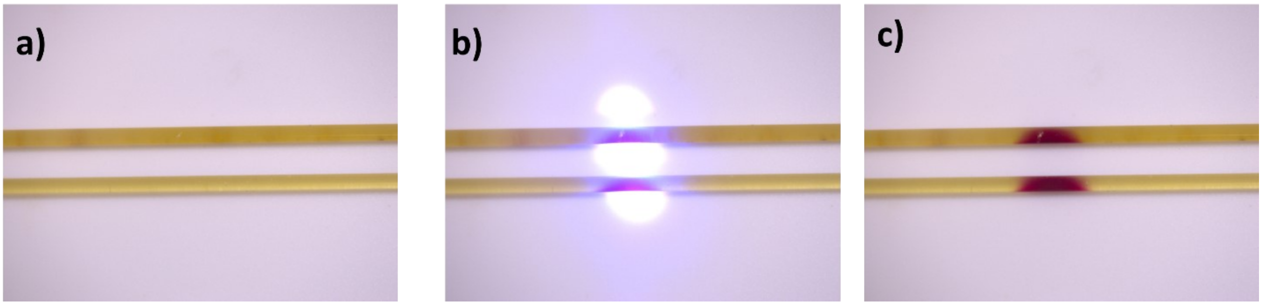

Figure S11: Effect of irradiation of 405 nm laser pointer light on a DBF solution of H_2bdc-dpq (upper tube) and on a solution containing both H_3hmtt and H_2bdc-dpq (lower tube) showing photochromism in both cases. **a)** before irradiation, **b)** with irradiation and **c)** after irradiation.

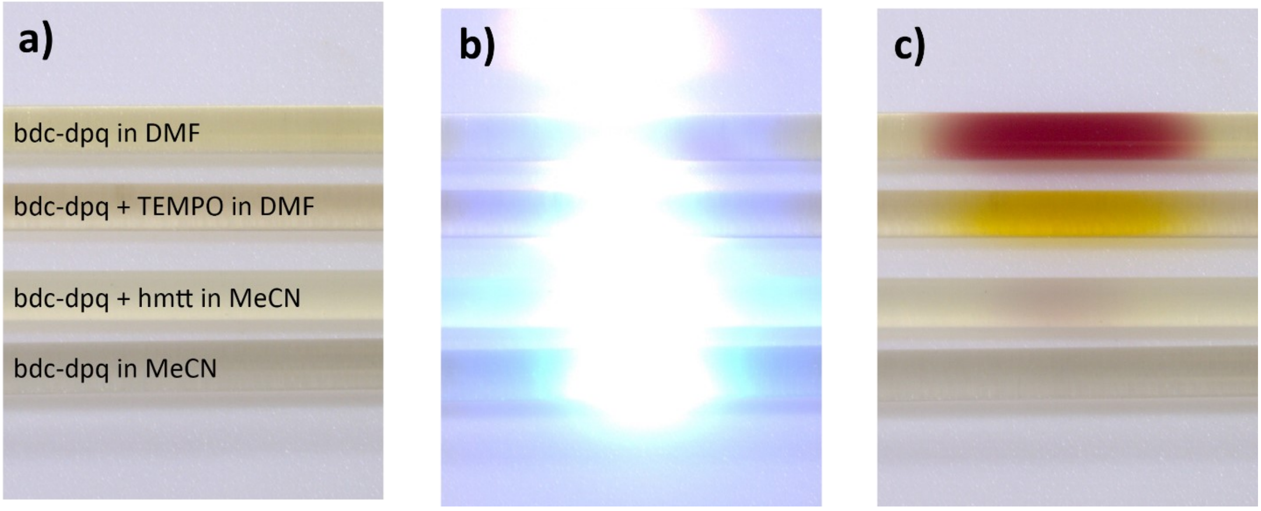

Figure S12: Effect of irradiation of 405 nm laser pointer light on a DMF and MeCN solution of H_2bdc-dpq, H_2bdc-dpq and the radical trap, TEMPO and on a solution containing both H_3hmtt and H_2bdc-dpq in MeCN, **a)** before irradiation, **b)** with irradiation and **c)** after irradiation. Photochromism is observed in only for H_2bdc-dpq in DMF and not in the other cases.

13. Electron Paramagnetic Resonance Spectroscopy

EPR spectra were measured BRUKER EMXmicro X-band spectrometer with a microwave power of 0.2 mW to avoid saturation effects. The measurements were performed using a Helium Oxford flow cryostat ESR900 and spectra were recorded at T = 100 K. g-value has been calculated using the following formula; $\boldsymbol{g = hv/B\mu_B}$; where $\boldsymbol{h}$ = Planck‘s constant, $\boldsymbol{v}$ – Microwave frequency, $\boldsymbol{B}$ – Magnetic field and $\boldsymbol{\mu_B}$ - Bohr magneton.

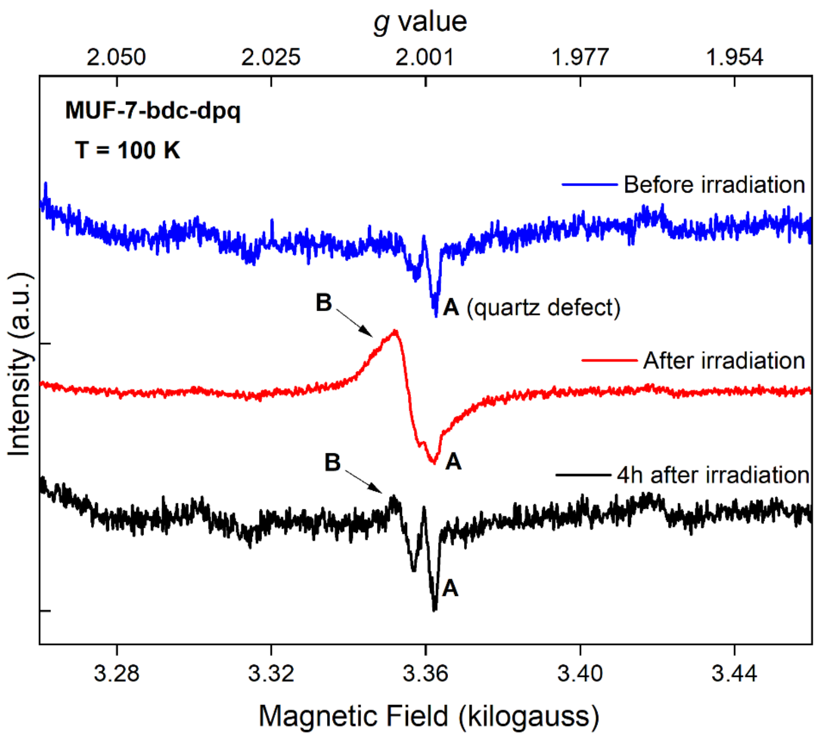

Figure S13: EPR spectrum of NC-MUF-7-bdc-dpq before irradiation (blue), immediately after irradiation (red), and 4 hours after irradiation (black). The signal marked **B** (g = 2.0037) is from organic radicals, which emerges after irradiation and drop in intensity 4 hours after irradiation. Signal A (g = 2.0008) is from a defect in the quartz sample tube.[4]

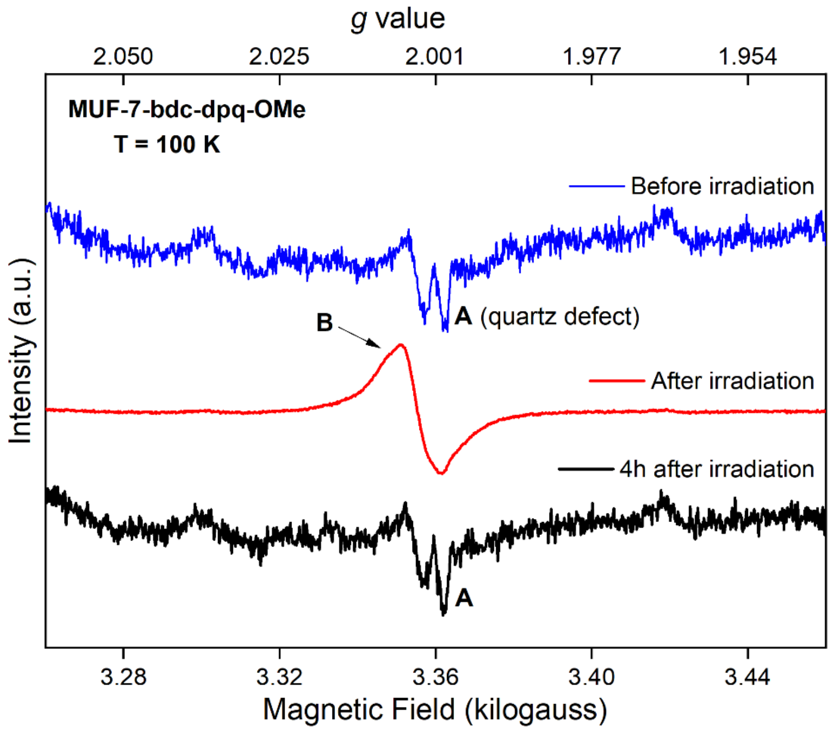

Figure S14: EPR spectrum of NC-MUF-7-bdc-dpq-OMe before irradiation (blue), immediately after irradiation (red), and 4 hours after irradiation (black). The signal marked **B** (g = 2.0037) is from organic radicals, which emerges after irradiation and drop in intensity 4 hours after irradiation. Signal **A** (g = 2.0008) is from a defect in the quartz sample tube.

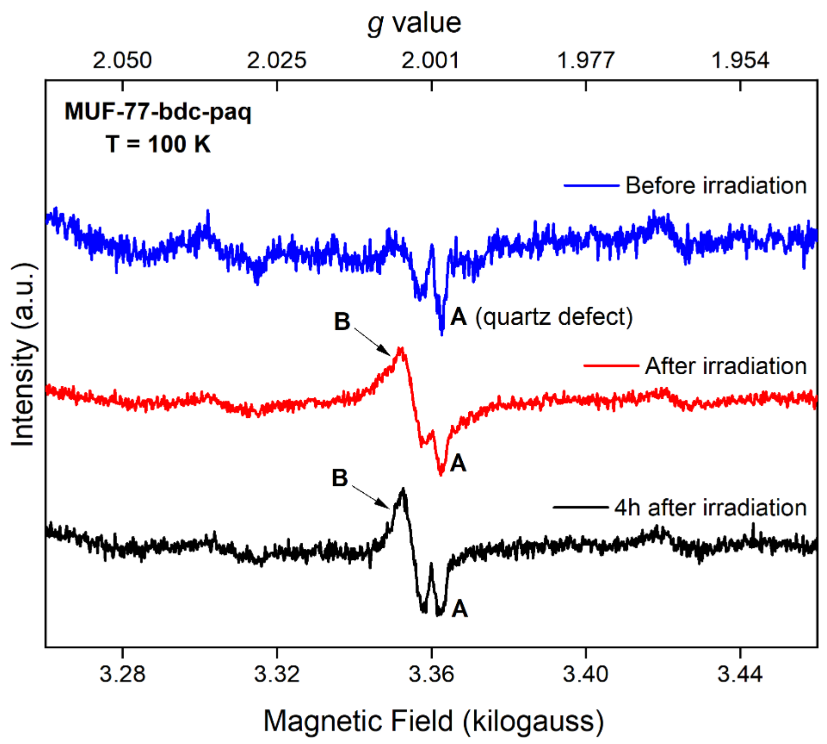

Figure S15: EPR spectrum of NC-MUF-77-bdc-paq before irradiation (blue), immediately after irradiation (red), and 4 hours after irradiation (black). The signal marked **B** (g = 2.0037) is from organic radicals, which emerges after irradiation and drop in intensity 4 hours after irradiation. Signal **A** (g = 2.0008) is from a defect in the quartz sample tube.

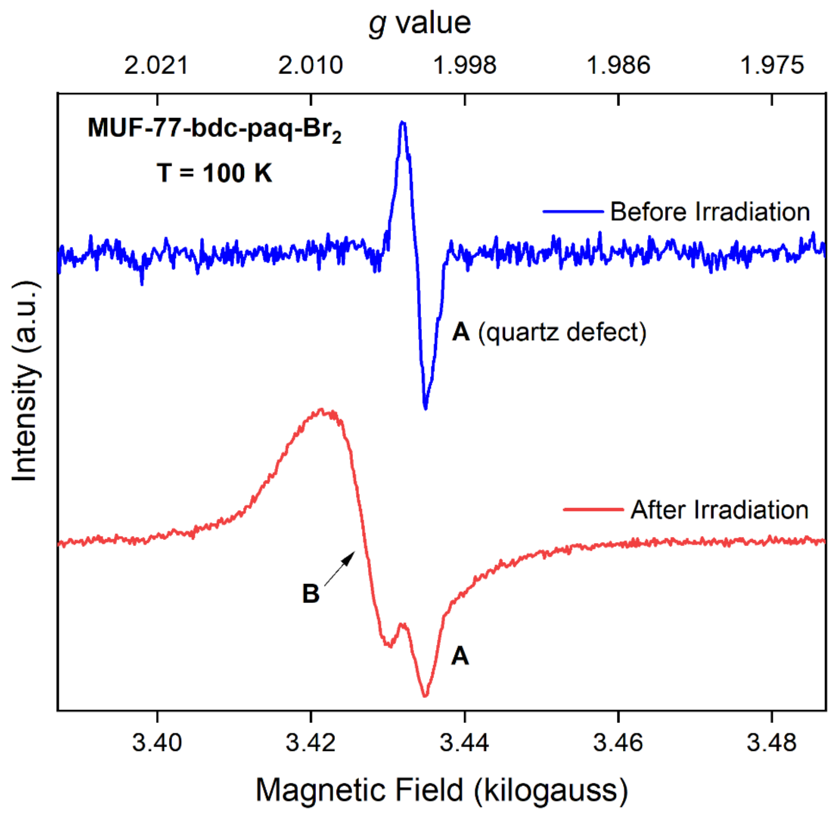

Figure S16: EPR spectrum of NC-MUF-77-bdc-paq-Br_2 before irradiation (blue), immediately after irradiation (red), and 4 hours after irradiation (black). The signal marked **B** (g = 2.0037) is from organic radicals, which emerges after irradiation. Signal **A** (g = 2.0008) is from a defect in the quartz sample tube.

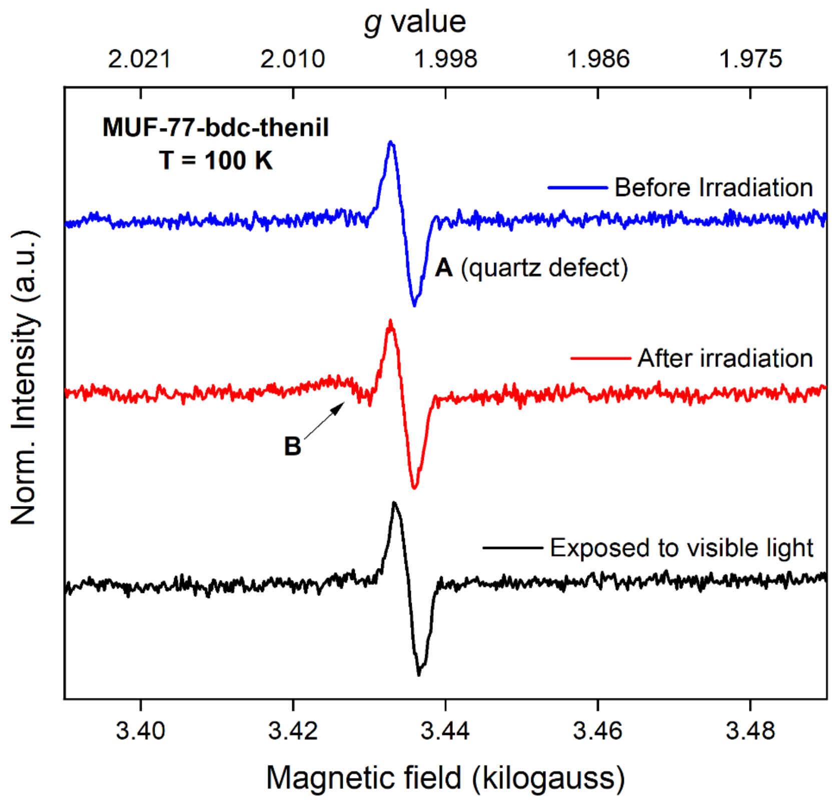

Figure S17: EPR spectrum of NC-MUF-77-bdc-thenil before irradiation (blue), immediately after irradiation (red), and exposed to visible light. The weak signal marked **B** (g = 2.0037) is from organic radicals, which emerges after irradiation. Signal A (g = 2.0008) is from a defect in the quartz sample tube.

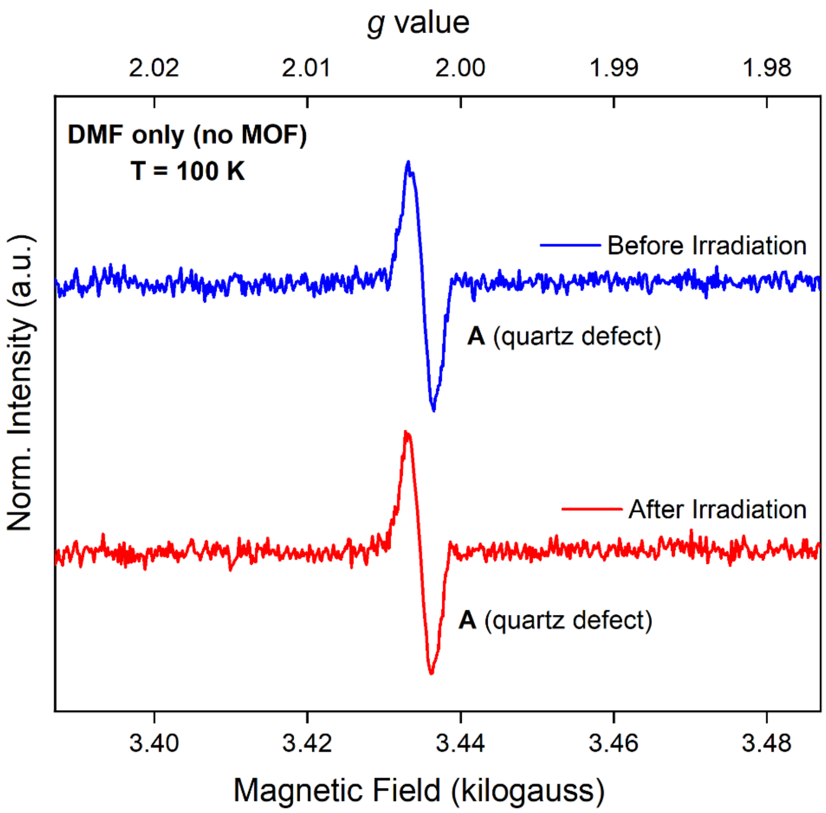

Figure S18: EPR spectrum of DMF before and after irradiation with a 405 nm laser pointer. Only signal **A** (g = 2.0008) is visible, which is assigned to a defect in the quartz sample tube.

14. UV-Visible Spectra of NC-MUF-7 and NC-MUF-77 suspensions

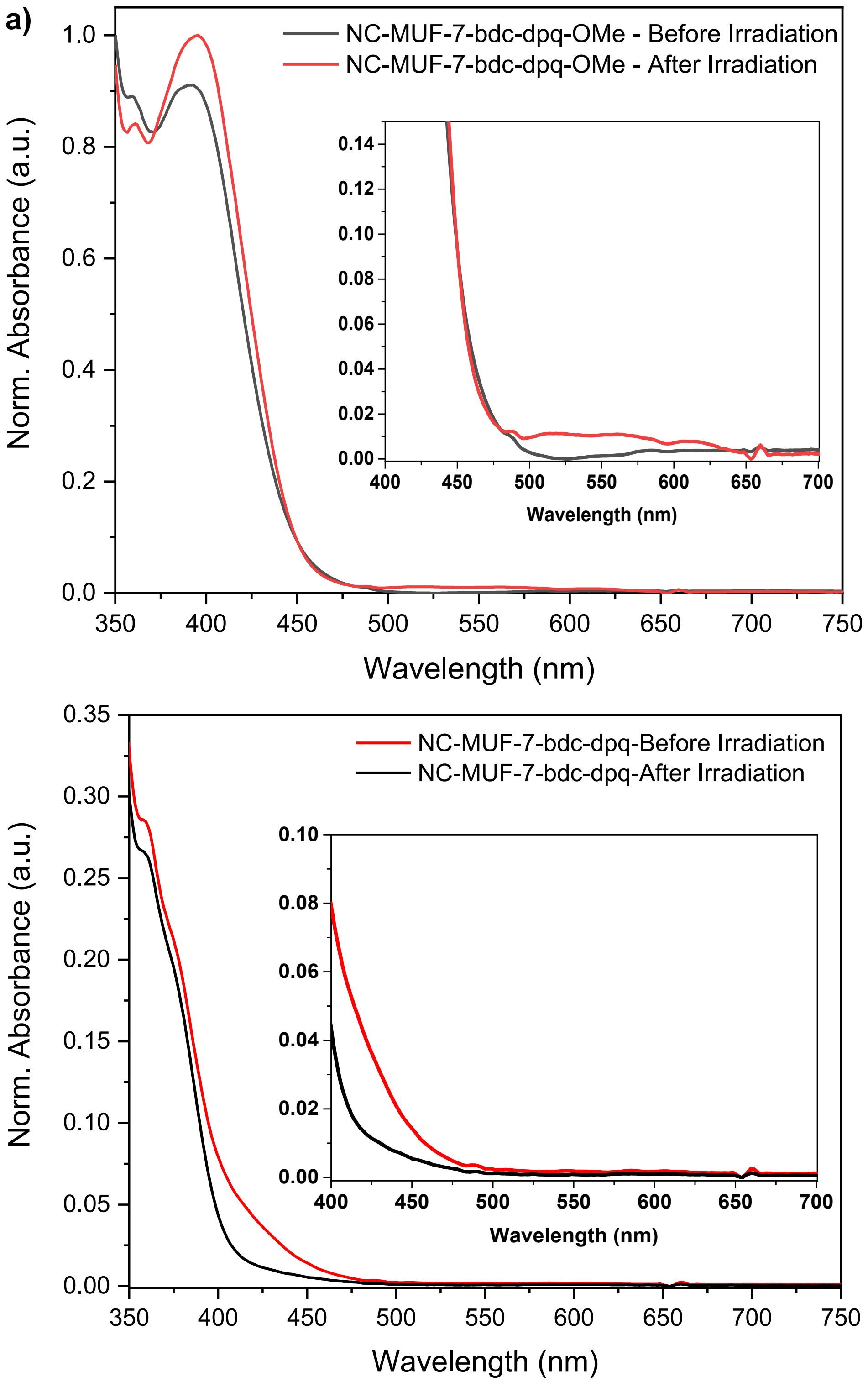

Figure S19: UV-Visible spectra of nanocrystalline MOF suspensions measured using CloudSpec **a)** NC-MUF-7-bdc-dpq-OMe **b)** NC-MUF-7-bdc-dpq.

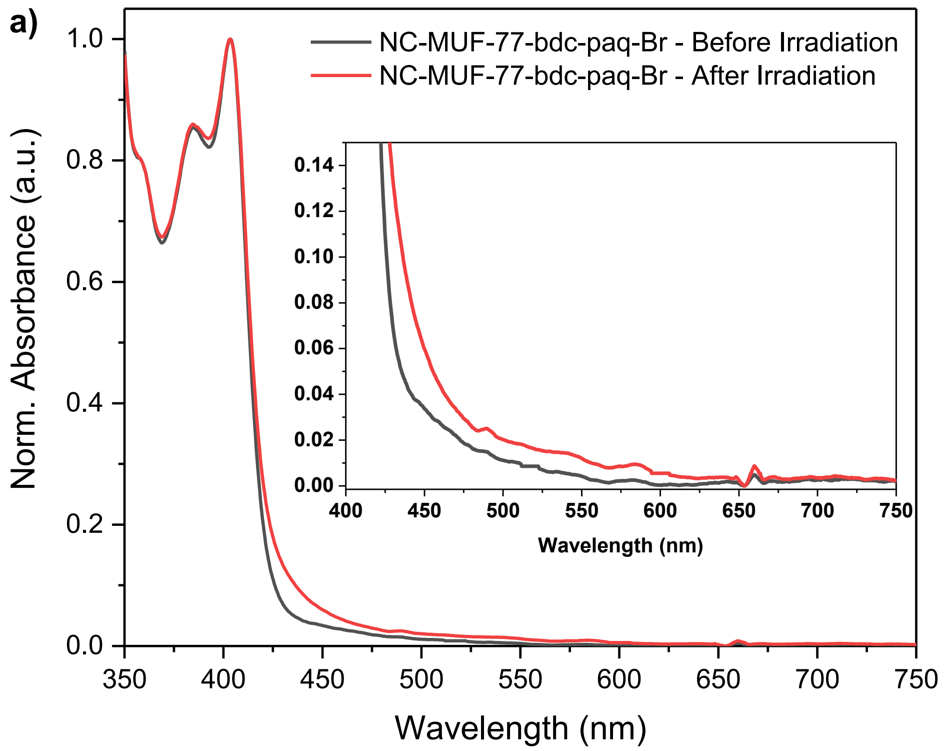

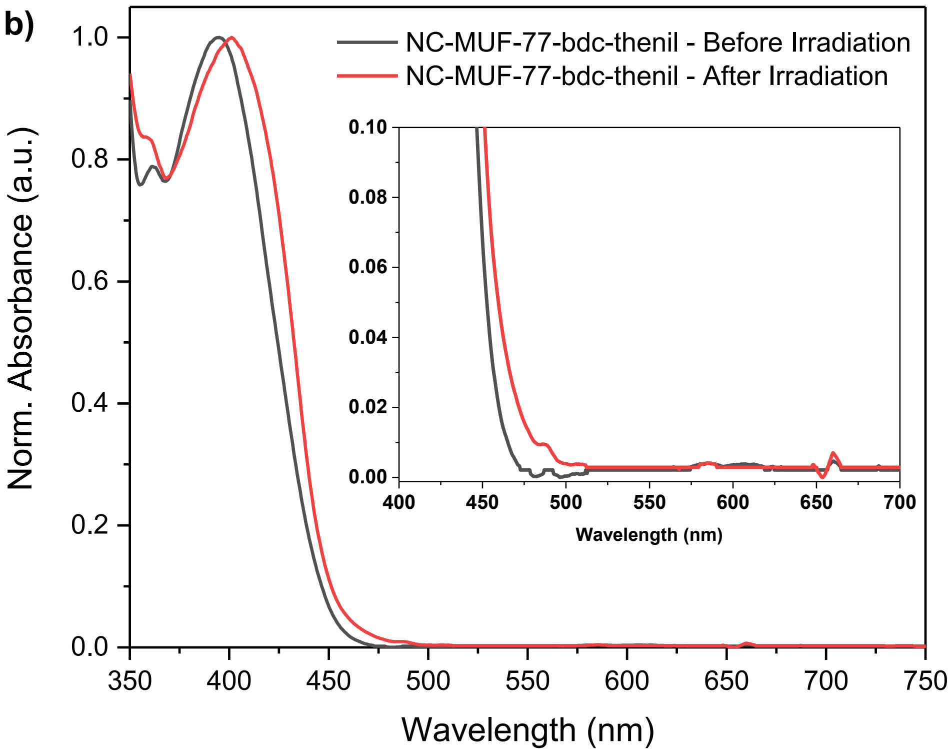

Figure S20: UV-Visible spectra of nanocrystalline MOF suspensions measured using CloudSpec **a)** NC-MUF-7-bdc-paq-Br **b)** NC-MUF-77-bdc-thenil.

15. Emission Spectra

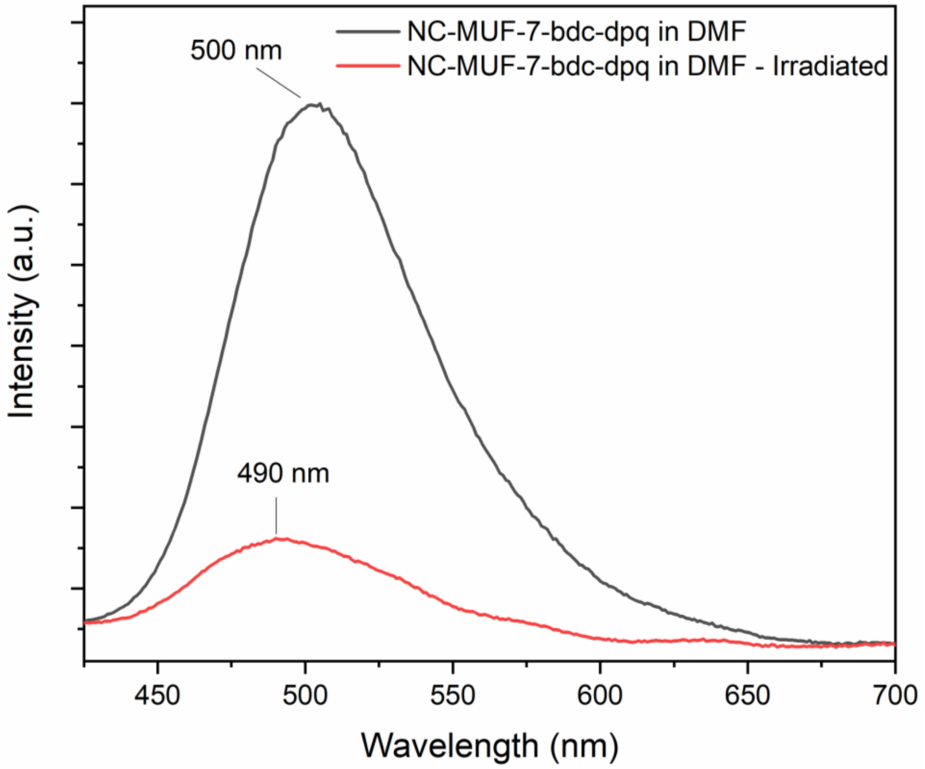

Figure S21: Emission spectra (λ_{ex} = 390 nm) of NC-MUF-7-bdc-dpq in DMF before and after irradiation.

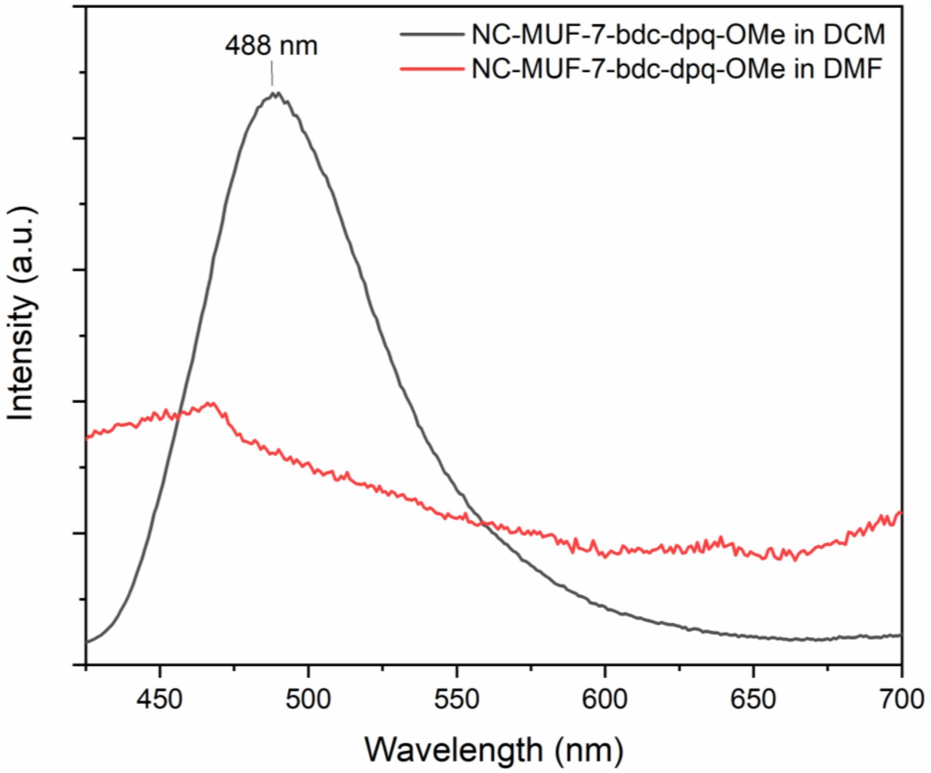

Figure S22: Emission spectra (λ_{ex} = 390 nm) of NC-MUF-7-bdc-dpq-OMe in dichloromethane (DCM) and DMF.

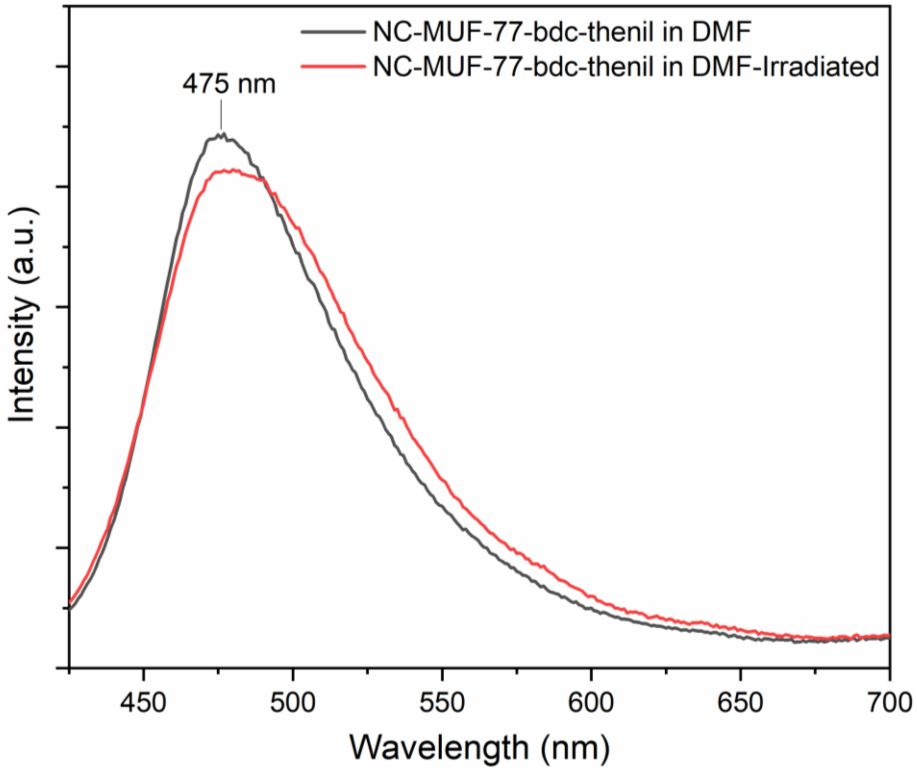

Figure S23: Emission spectra (λ_{ex} = 390 nm) of NC-MUF-77-bdc-thenil in DMF before and after irradiation.

16. Crystallographic details and bond length analysis

X-ray diffraction data were collected using a Bruker D8 Venture diffractometer with Cu_α radiation (wavelength = 1.54180 Å), with a diamond microfocus X-ray source and a Photon III 28 detector. For SCXRD, the crystals were exchanged with *N*,*N*-dibutylformamide (DBF) and mounted on a nylon loop using paratone oil. The crystal was kept under an atmosphere of nitrogen at 293 K for all measurements. For irradiation, a portable UV lamp was used, and the sample was kept irradiated throughout the measurement.

The data were integrated using APEX3 and processed in OLEX2 using SHELXL for refinement.[5,6] The Solvent Mask function was used for some structures to find the number of unallocated electrons which were used to compare to occluded DBF molecules.

Table S3: Crystallographic details for MUF-7-bdc-dpq before irradiation and while being irradiated with UV light.

**Identification code**	MUF-7-**bdc-dpq**-<br>before irradiation<br>CCDC 2189834	MUF-7-**bdc-dpq**-<br>with irradiation<br>CCDC 2189835
Empirical formula	$C_{66}H_{46}NO_{13}Zn_4$	$C_{66}H_{46}NO_{13}Zn_4$
Formula weight	1322.52	1322.52
Temperature/K	293.0	293.0
Crystal system	cubic	cubic
Space group	I-43d	I-43d
a/Å	59.5745(16)	59.485(2)
b/Å	59.5745(16)	59.485(2)
c/Å	59.5745(16)	59.485(2)
α/°	90	90
β/°	90	90
γ/°	90	90
Volume/Å^3	211437(17)	210488(21)
Z	48	48
ρ_{calc} / g cm^{-3}	0.498	0.501
μ/mm^{-1}	0.798	0.804
F(000)	32304.0	32304.0
Wavelength	CuKα (λ = 1.54178 nm)	CuKα (λ = 1.54178 nm)
Data range for refinement/°	3.632 to 116.318	3.638 to 108.544
Reflections collected	278343	342112
Independent reflections	24559 [R_{int} = 0.0420, R_{sigma} = 0.0251]	21435 [R_{int} = 0.0538, R_{sigma} = 0.0248]
Data/restraints/parameters	24559/504/764	21435/505/753
Goodness-of-fit on F^2	1.143	1.414
Final R indexes [I>=2σ (I)]	R_1 = 0.0788, wR_2 = 0.2233	R_1 = 0.1091, wR_2 = 0.2961
Final R indexes [all data]	R_1 = 0.0867, wR_2 = 0.2336	R_1 = 0.1141, wR_2 = 0.3044
Largest diff. peak/hole / e Å^{-3}	1.10/-0.52	1.52/-0.61
Flack parameter	0.5	0.5

Code: - MUF-7-**bdc-dpq**-before irradiation: JC286_dpq_bare_6
- MUF-7-**bdc-dpq**-with irradiation: JC286_dpq_UV_6

Table S4: Crystallographic details for MUF-7-bdc-dpq-OMe before irradiation and while being irradiated with UV light.

**Identification code**	MUF-7-**bdc-dpq-OMe** before irradiation CCDC 2189836	MUF-7-**bdc-dpq-OMe** with irradiation CCDC 2189833
Empirical formula	$C_{67}H_{48}NO_{14}Zn_4$	$C_{67}H_{48}NO_{14}Zn_4$
Formula weight	1352.54	1352.54
Temperature/K	293.0	293.0
Crystal system	cubic	cubic
Space group	I-43d	I-43d
a/Å	59.8236(17)	59.551(4)
b/Å	59.8236(17)	59.551(4)
c/Å	59.8236(17)	59.551(4)
α/°	90	90
β/°	90	90
γ/°	90	90
Volume/Å^3	214100(19)	211186(47)
Z	48	48
ρ_{calc} / g cm^{-3}	0.504	0.510
μ/mm^{-1}	0.799	0.810
F(000)	33072.0	33072.0
Radiation	CuKα (λ = 1.54178 nm)	CuKα (λ = 1.54178 nm)
Data range for refinement/°	3.618 to 106.97	3.634 to 117.078
Reflections collected	177251	162081
Independent reflections	21077 [R_{int} = 0.0517, R_{sigma} = 0.0399]	24402 [R_{int} = 0.0582, R_{sigma} = 0.0458]
Data/restraints/parameters	21077/18/748	24402/0/784
Goodness-of-fit on F^2	1.111	1.594
Final R indexes [I>=2σ (I)]	R_1 = 0.0855, wR_2 = 0.2423	R_1 = 0.1290, wR_2 = 0.3532
Final R indexes [all data]	R_1 = 0.0968, wR_2 = 0.2574	R_1 = 0.1605, wR_2 = 0.3833
Largest diff. peak/hole / e Å^{-3}	0.84/-0.67	1.32/-0.66
Flack parameter	0.5	0.5

Code: - MUF-7-**bdc-dpq-OMe**-before irradiation: JC5286_OCH3_0m
- MUF-7-**bdc-dpq-OMe** with irradiation: JC5296_OMe_UV_N_2

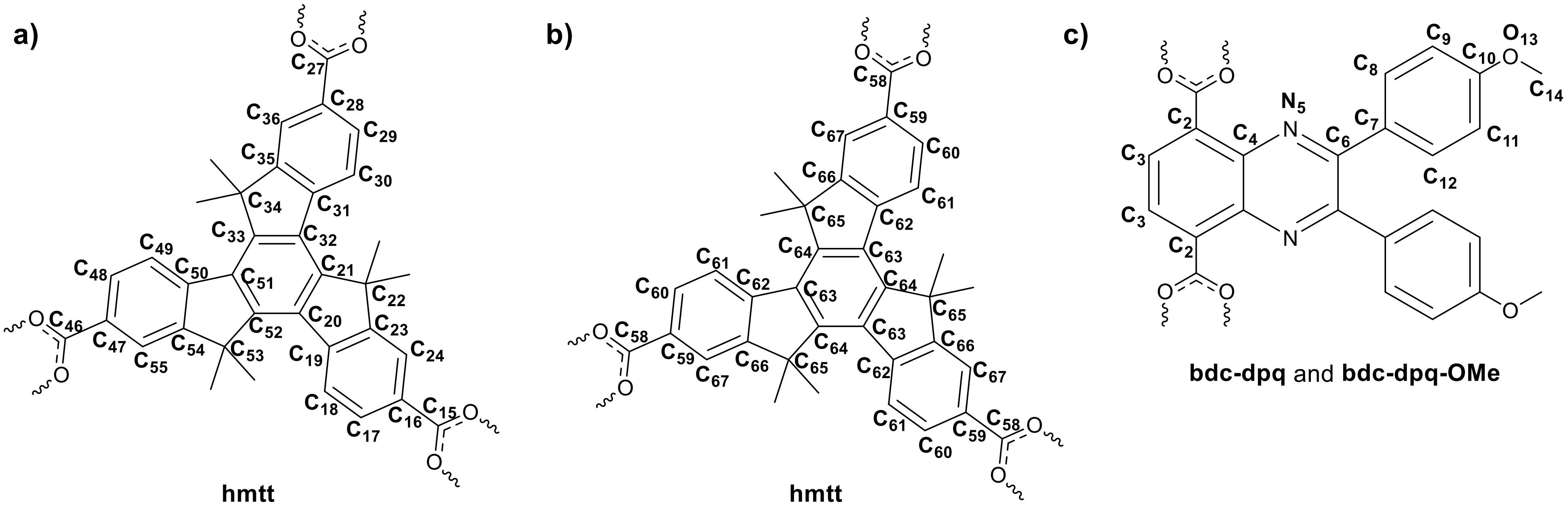

Figure S24: Atom labels used for the ligands in the SCXRD structures of MUF-7-bdc-dpq and MUF-7-bdc-dpq-OMe. Note that there are two non-equivalent hmtt ligands **a)** without C_3 symmetry and **b)** with C_3 symmetry. **c)** Labels for the bdc-dpq and bdc-dpq-OMe ligands.

Table S5: Selected bond lengths of MUF-7-bdc-dpq without and with 405 nm radiation as determined by X-ray crystallography. Atom labels are defined in Figure S18 and distances are given in Å.

Bond	Length without irradiation	Length with irradiation	Bond length change	Bond	Length without irradiation	Length with irradiation	Bond length change
	**bdc-dpq**				**hmtt**		
C_1–C_2	1.523(10)	1.494(14)	–	C_{15} – C_{16}	1.501(12)	1.484(17)	–
C_2–C_3	1.365(12)	1.396(17)	+0.031	C_{16} – C_{17}	1.377(13)	1.382(17)	–
C_3–C_3	1.398(16)	1.33(3)	-0.068	C_{19} – C_{20}	1.497(13)	1.516(19)	–
C_2–C_4	1.393(12)	1.398(16)	–	C_{21} – C_{22}	1.559(13)	1.60(2)	+0.041
C_4 –C_4	1.420(14)	1.41(2)	–	C_{23} – C_{24}	1.361(13)	1.342(19)	–
C_4–N_5	1.369(10)	1.370(13)	–	C_{20} – C_{52}	1.410(14)	1.401(19)	–
N_5–C_6	1.308(10)	1.318(14)	–	C_{16} – C_{24}	1.377(13)	1.361(18)	–
C_6 –C_6	1.443(15)	1.48(2)	+0.037	C_{29} – C_{30}	1.401(16)	1.38(2)	–
C_6–C_7	1.489(11)	1.450(16)	-0.039	C_{27} – C_{28}	1.516(14)	1.44(2)	-0.076
C_7–C_8	1.404(12)	1.371(16)	-0.033	C_{21} – C_{32}	1.420(14)	1.39(2)	-0.03
C_8–C_9	1.381(13)	1.374(19)	–	C_{32} – C_{33}	1.387(14)	1.44(2)	-0.053
C_9–C_{10}	1.356(15)	1.356(19)	–	C_{31} – C_{32}	1.463(13)	1.45(2)	–
C_{10}–C_{11}	1.376(16)	1.39(2)	–	C_{46} – C_{47}	1.522(13)	1.449(19)	-0.073
C_{11}–C_{12}	1.393(14)	1.369(19)	-0.024	C_{47} – C_{48}	1.383(15)	1.39(2)	–
C_7–C_{12}	1.400(13)	1.405(18)	–	C_{54} – C_{55}	1.390(13)	1.352(17)	-0.038
				C_{50} – C_{51}	1.487(13)	1.45(2)	–
				C_{51} – C_{52}	1.426(14)	1.39(2)	-0.036
				C_{52} – C_{53}	1.506(13)	1.56(2)	+0.054
				C_{53} – C_{54}	1.512(13)	1.50(2)	–
				C_{58} – C_{59}	1.501(11)	1.489(15)	–
				C_{60} – C_{61}	1.381(3)	1.390(19)	–
				C_{61} – C_{62}	1.398(13)	1.370(18)	-0.028
				C_{64} – C_{65}	1.541(12)	1.551(17)	–
				C_{63} – C_{64}	1.406(12)	1.443(18)	+0.037
				C_{63} – C_{64}	1.385(13)	1.373(18)	-0.026
				C_{20} – C_{21}	1.364(14)	1.35(2)	–
				C_{33} – C_{51}	1.403(14)	1.46(2)	+0.057

Table S6: Selected bond lengths of ligands in MUF-7-bdc-dpq-OMe without and with 405 nm irradiation as determined by X-ray crystallography. Atom labels are defined in Figure S18 and distances are given in Å.

Bond	Length without irradiation	Length with irradiation	Bond length change	Bond	Length without irradiation	Length with irradiation	Bond length change
bdc-dpq-OMe				hmtt			
C_1 – C_2	1.504(15)	1.554(18)	+0.05	C_{15} – C_{16}	1.465(16)	1.49(2)	–
C_2 – C_3	1.333(18)	1.35(2)	–	C_{16} – C_{17}	1.391(17)	1.38(3)	–
C_3 – C_3	1.42(2)	1.37(2)	-0.05	C_{19} – C_{20}	1.503(16)	1.50(3)	–
C_2 – C_4	1.407(17)	1.45(2)	+0.043	C_{21} – C_{22}	1.520(15)	1.52(3)	–
C_4 – C_4	1.41(2)	1.38(3)	–	C_{23} – C_{24}	1.391(16)	1.37(2)	–
C_4 – N_5	1.396(15)	1.38(2)	–	C_{20} – C_{52}	1.392(14)	1.37(3)	–
N_5 – C_6	1.323(16)	1.38(2)	+0.057	C_{20} – C_{21}	1.403(16)	1.46(2)	+0.06
C_6 – C_6	1.44(2)	1.38(3)	-0.06	C_{31} – C_{32}	1.499(11)	1.48(2)	–
C_6 – C_7	1.453(17)	1.46(2)	–	C_{32} – C_{33}	1.416(16)	1.41(2)	–
C_7 – C_8	1.423(19)	1.36(3)	-0.06	C_{21} – C_{32}	1.388(16)	1.31(3)	-0.078
C_8 – C_9	1.397(18)	1.42(2)	–	C_{46} – C_{47}	1.455(13)	1.47(3)	–
C_9 – C_{10}	1.352(18)	1.40(3)	+0.048	C_{50} – C_{51}	1.505(11)	1.45(2)	-0.055
C_{10} – C_{11}	1.380(18)	1.39(3)	–	C_{51} – C_{52}	1.397(17)	1.45(3)	-0.053
C_{10} – O_{13}	1.327(15)	1.36(2)	+0.033	C_{52} – C_{53}	1.570(16)	1.51(3)	-0.06
O_{13} – C_{14}	1.40(2)	1.43(3)	–	C_{53} – C_{54}	1.510(15)	1.54(3)	–
C_{11} – C_{12}	1.385(17)	1.42(2)	+0.035	C_{58} – C_{59}	1.530(15)	1.48(2)	-0.05
C_7 – C_{12}	1.407(17)	1.39(2)	–	C_{60} – C_{61}	1.382(17)	1.33(3)	+0.052
				C_{62} – C_{63}	1.514(15)	1.56(3)	+0.046
				C_{61} – C_{62}	1.376(17)	1.42(3)	+0.044
				C_{64} – C_{65}	1.574(16)	1.49(3)	-0.084
				C_{66} – C_{67}	1.396(16)	1.36(2)	+0.071

Table S7: Crystallographic details for MUF-77-bdc-paq-Br without irradiation.

**Identification code**	MUF-77-bdc-paq-Br<br>CCDC 2191826
Empirical formula	$C_{66}H_{44}BrNO_{13}Zn_4$
Formula weight	1400.41
Temperature/K	297.0
Crystal system	cubic
Space group	Pm-3
a/Å	29.9583(5)
b/Å	29.9583(5)
c/Å	29.9583(5)
α/°	90
β/°	90
γ/°	90
Volume/Å^3	26887.6(13)
Z	6
ρ_{calc} / g cm^{-3}	0.519
μ/mm^{-1}	1.050
F(000)	4236.0
Radiation	CuKα (λ = 1.54178 nm)
Data range for refinement/°	4.17 to 106.85
Reflections collected	23386
Independent reflections	5502 [R_{int} = 0.0297, R_{sigma} = 0.0248]
Data/restraints/parameters	5502/0/280
Goodness-of-fit on F^2	1.094
Final R indexes [I>=2σ (I)]	R_1 = 0.0474, wR_2 = 0.1578
Final R indexes [all data]	R_1 = 0.0506, wR_2 = 0.1616
Largest diff. peak/hole / e Å^{-3}	0.39/-0.35

17. ^{1}H NMR spectra of digested MOFs

The following protocol was used for digestion of the MOFs for ^{1}H NMR spectroscopy: The MOF sample was washed and centrifuged five times with dry CH_2Cl_2 or acetone, then desolvated *in vacuo*. 0.60 mL of DCl/DMSO-d_6 (10 μL/600 μL) solution was used to digest around 3 mg of MOF. A ^{1}H NMR spectrum was acquired of the resulting clear solution of the dissolved framework.

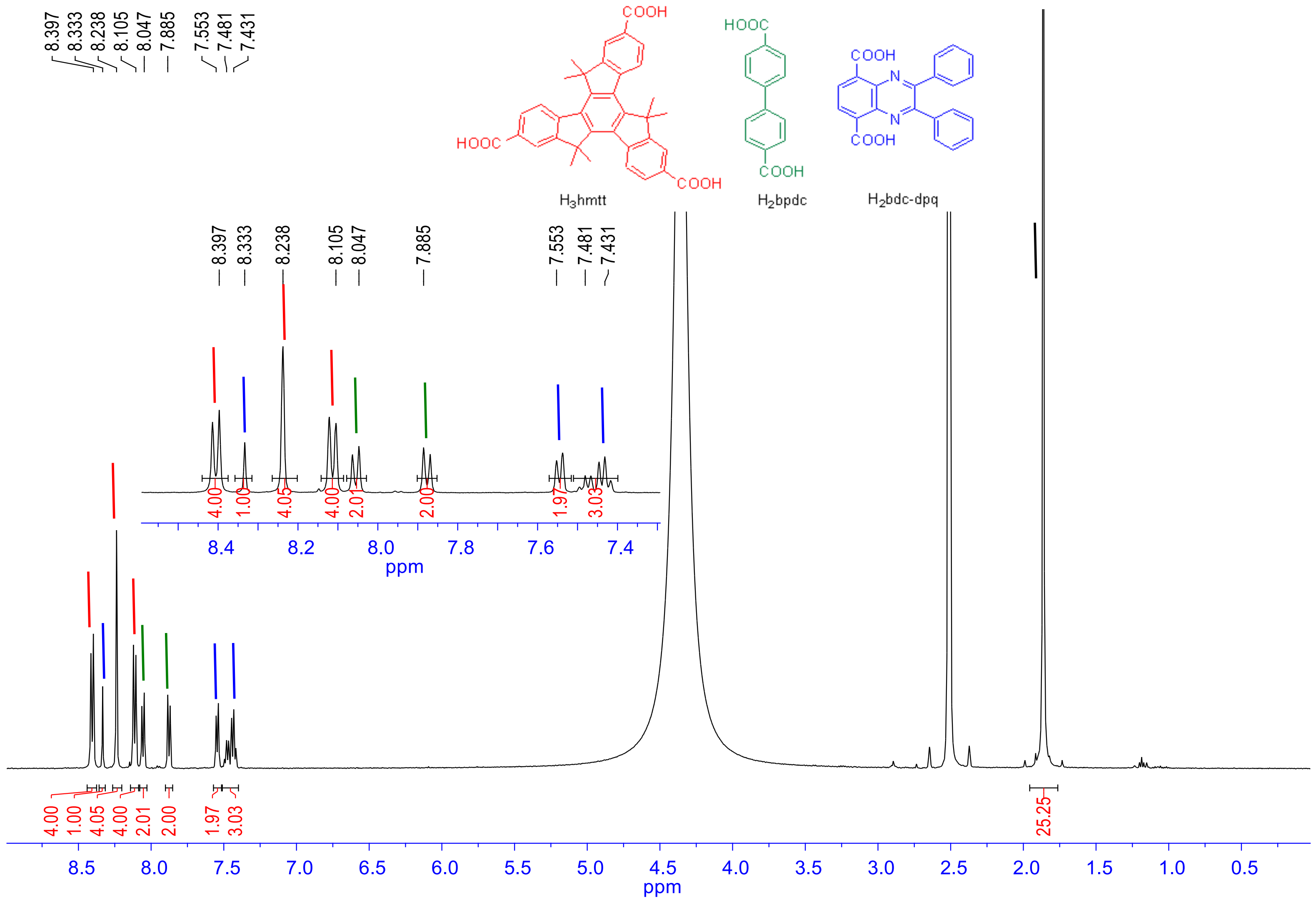

Figure S25: ^{1}H NMR spectrum of digested MUF-7-bdc-dpq, $[Zn_4O(hmtt)_{4/3}(bpdc)_{1/2}(bdc\text{-}dpq)_{1/2}]$ showing integrals that match with the formula.

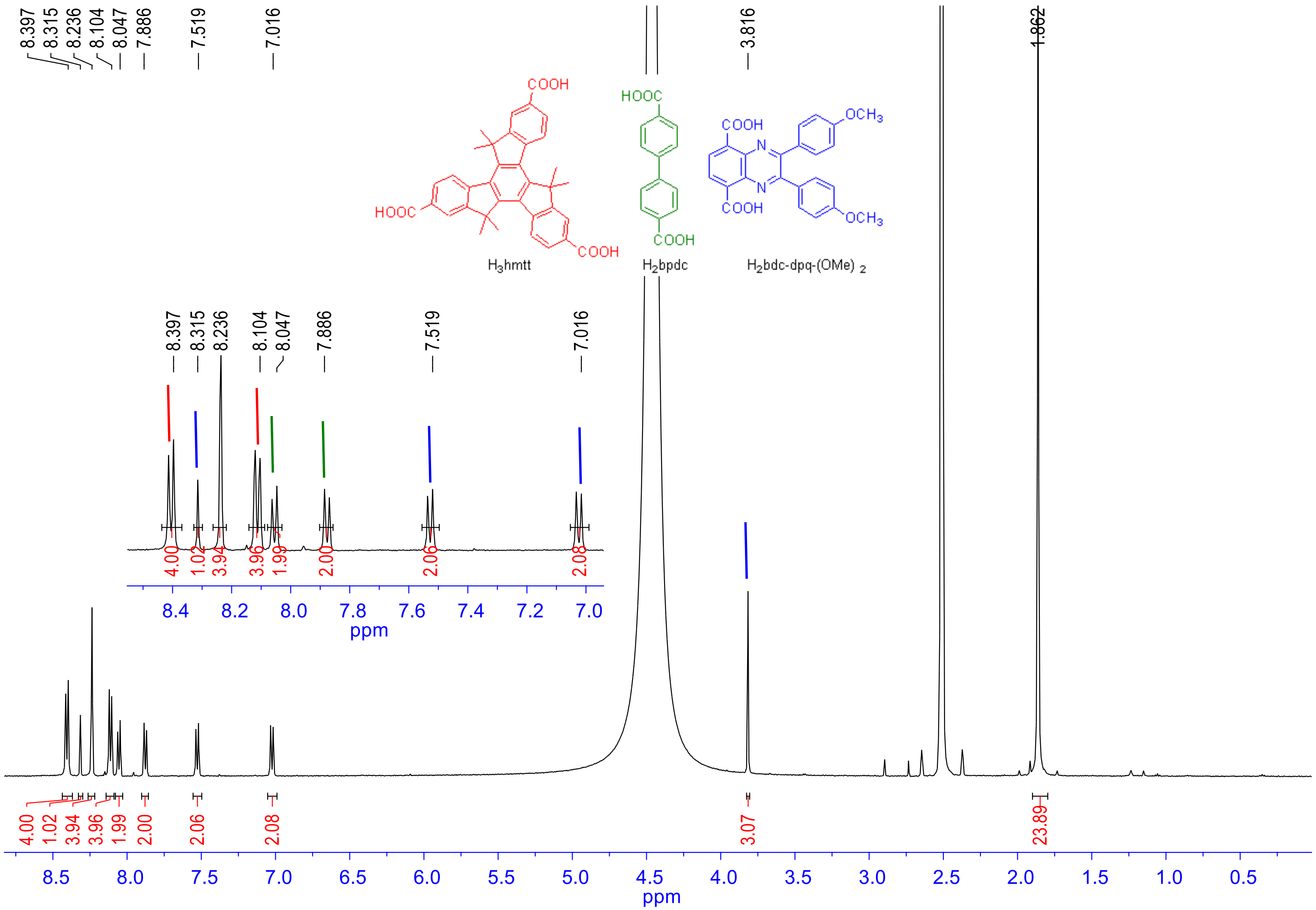

Figure S26: ^{1}H NMR spectrum of digested MUF-7-bdc-dpq-OMe, $[Zn_4O(hmtt)_{4/3}(bpdc)_{1/2}(bdc\text{-}dpq\text{-}OMe)_{1/2}]$ showing integrals that match with the formula.

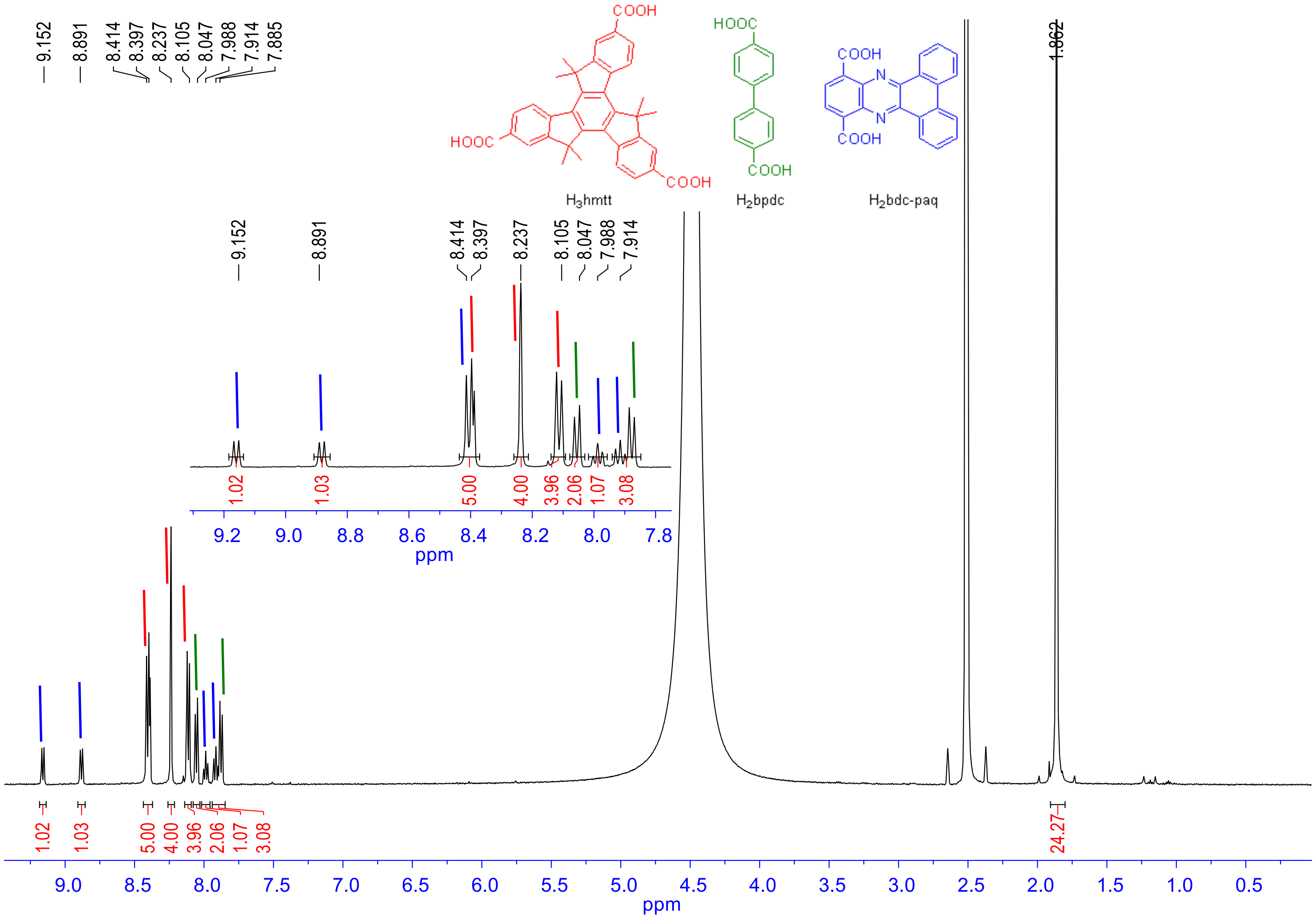

Figure S27: ^{1}H NMR spectrum of digested MUF-77-bdc-paq, [$Zn_4O(hmtt)_{4/3}(bpdc)_{1/2}(bdc\text{-}paq)_{1/2}$] showing integrals that match with the formula.

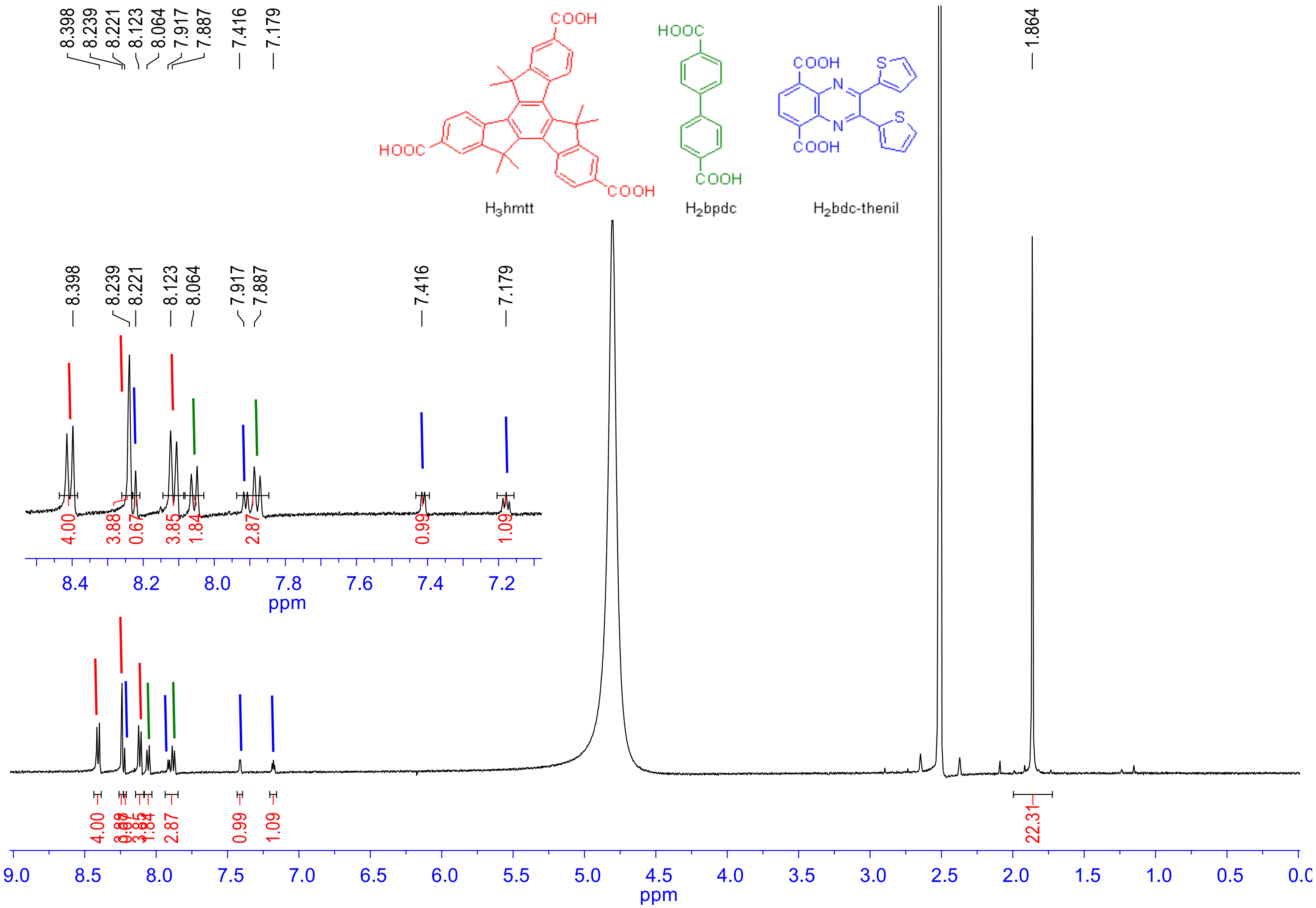

Figure S28: ^{1}H NMR spectrum of digested MUF-77-bdc-thenil, [$Zn_4O(hmtt)_{4/3}(bpdc)_{1/2}(bdc\text{-}thenil)_{1/2}$] showing integrals that match with the formula.

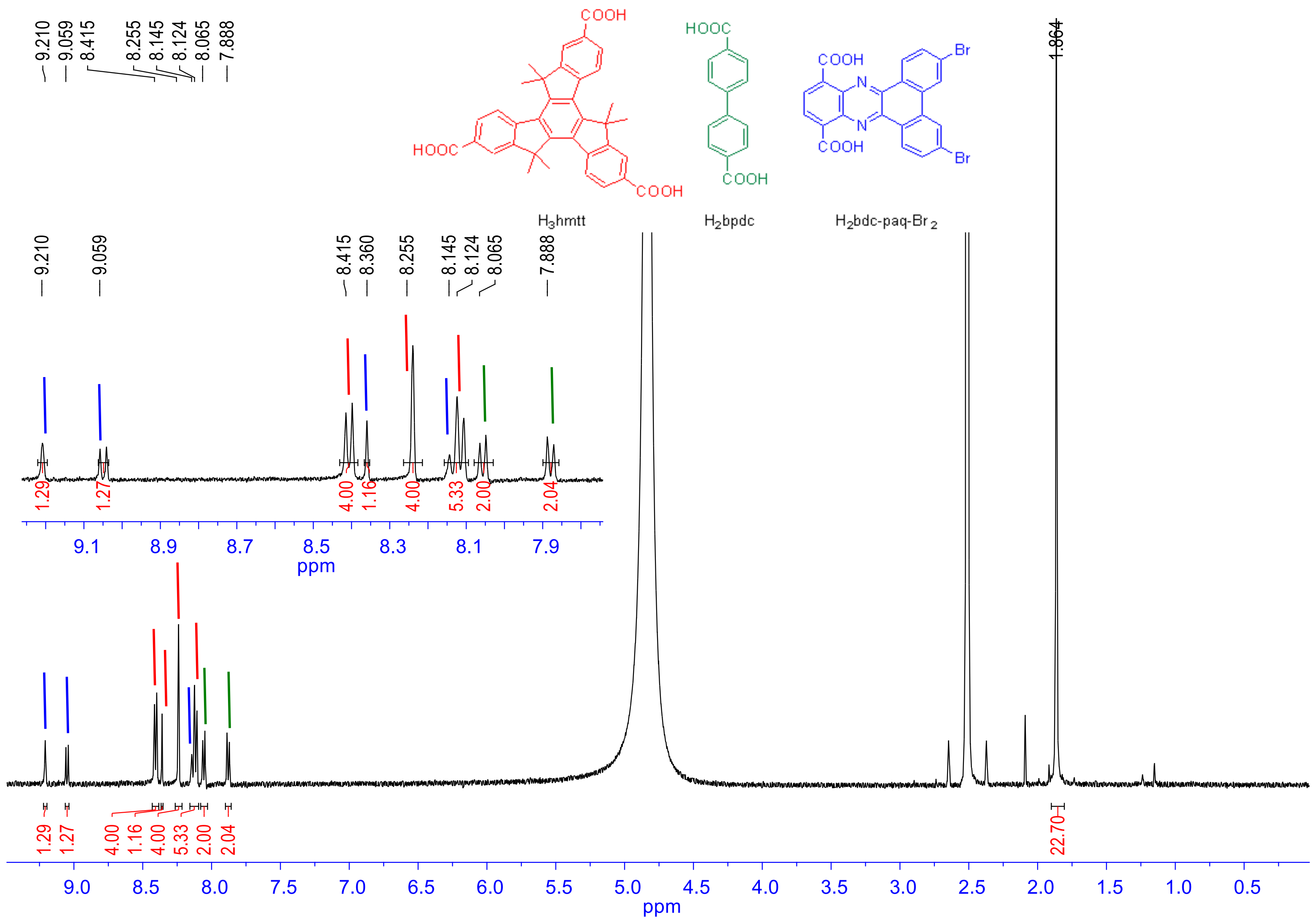

Figure S29: ^{1}H NMR spectrum of digested MUF-77-bdc-paq-Br$_2$, [$Zn_4O(hmtt)_{4/3}(bpdc)_{1/2}(bdc\text{-}paq\text{-}Br)_{1/2}$] showing integrals that match with the formula.

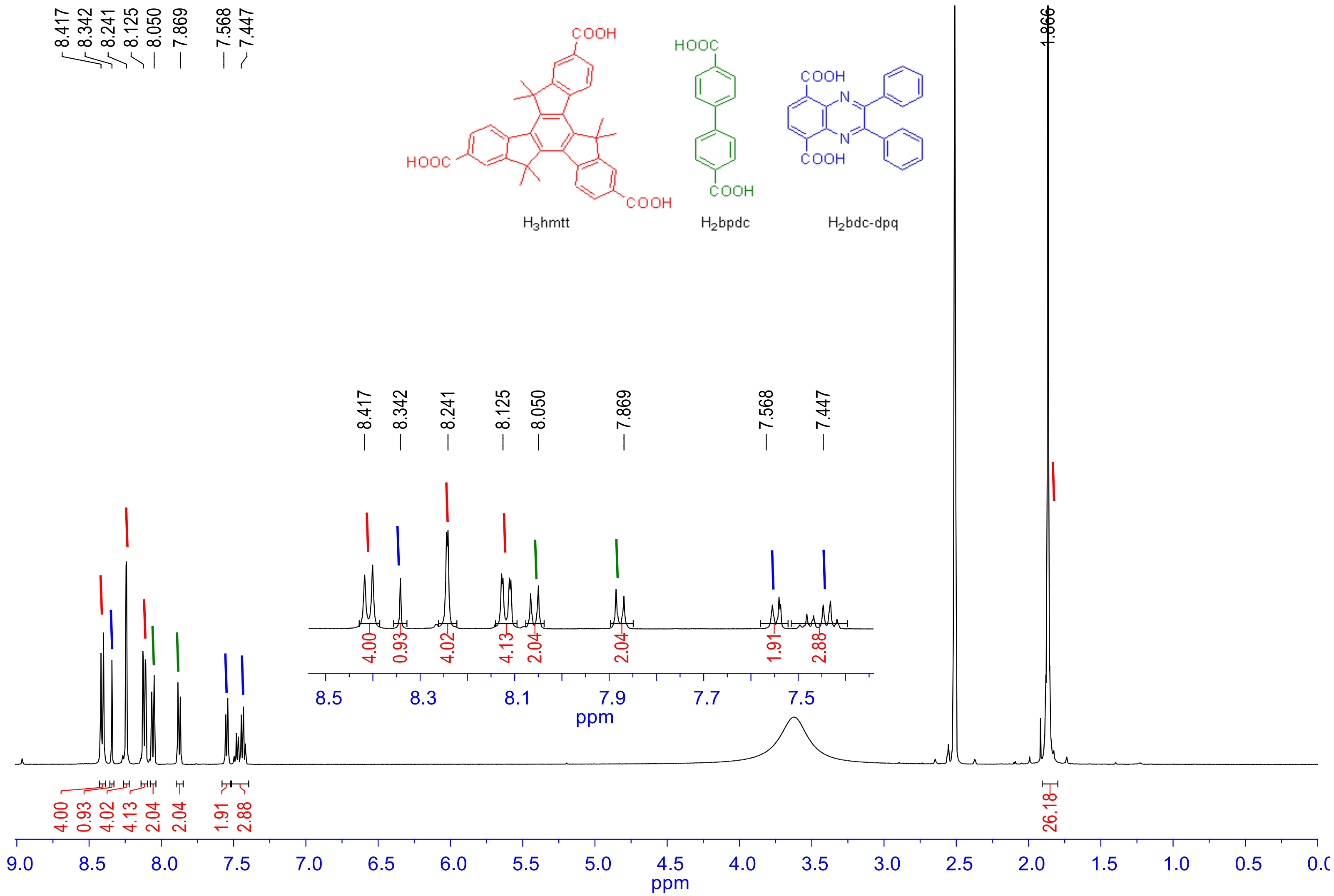

Figure S30: ^{1}H NMR spectrum of digested NC-MUF-7-bdc-dpq, [$Zn_4O(hmtt)_{4/3}(bpdc)_{1/2}(bdc\text{-}dpq)_{1/2}$] showing integrals that match with the formula.

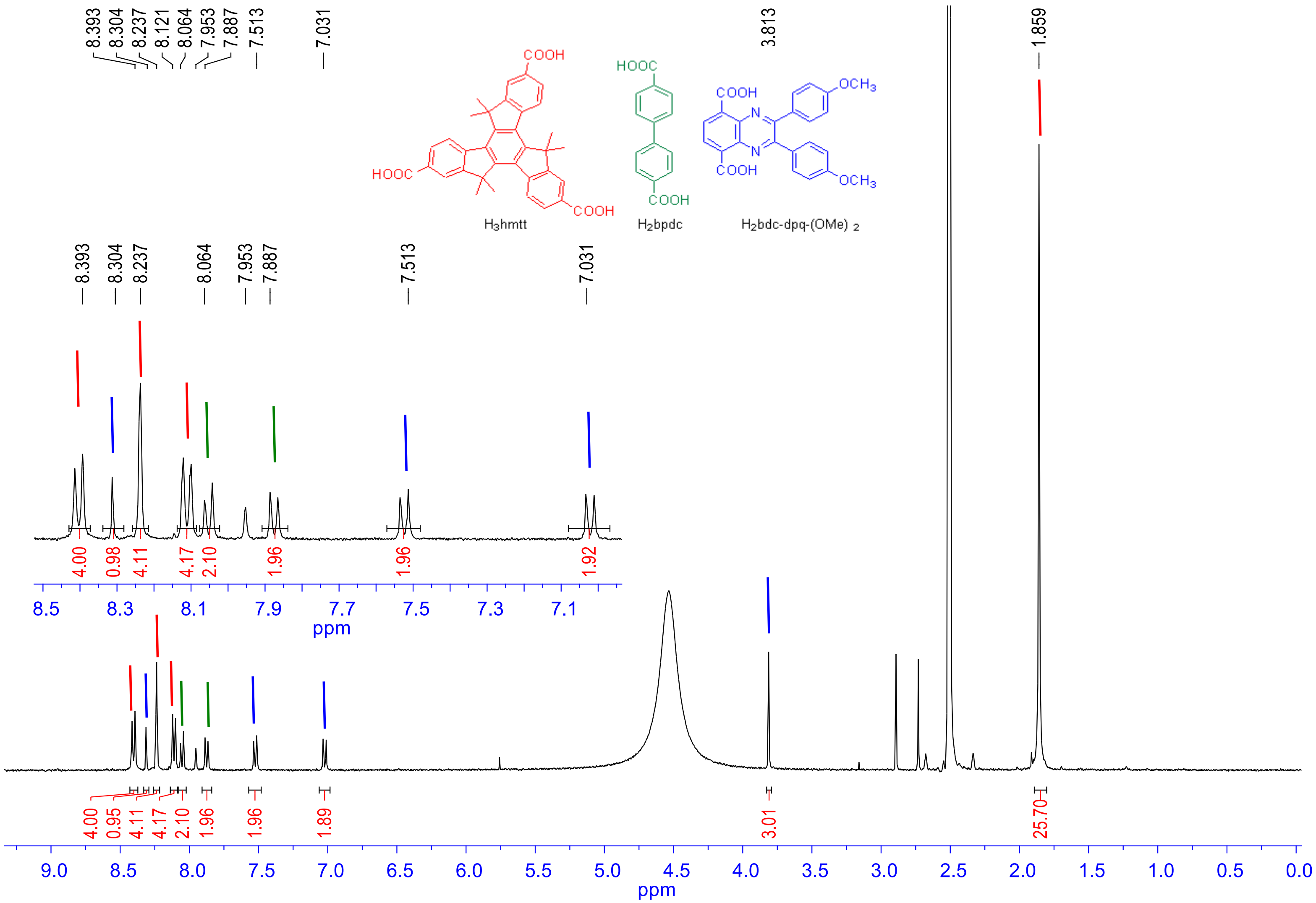

Figure S31: ^{1}H NMR spectrum of digested NC-MUF-77-bdc-dpq-OMe, $[Zn_4O(hmtt)_{4/3}(bpdc)_{1/2}(bdc\text{-}dpq\text{-}OMe)_{1/2}]$ showing integrals that match with the formula.

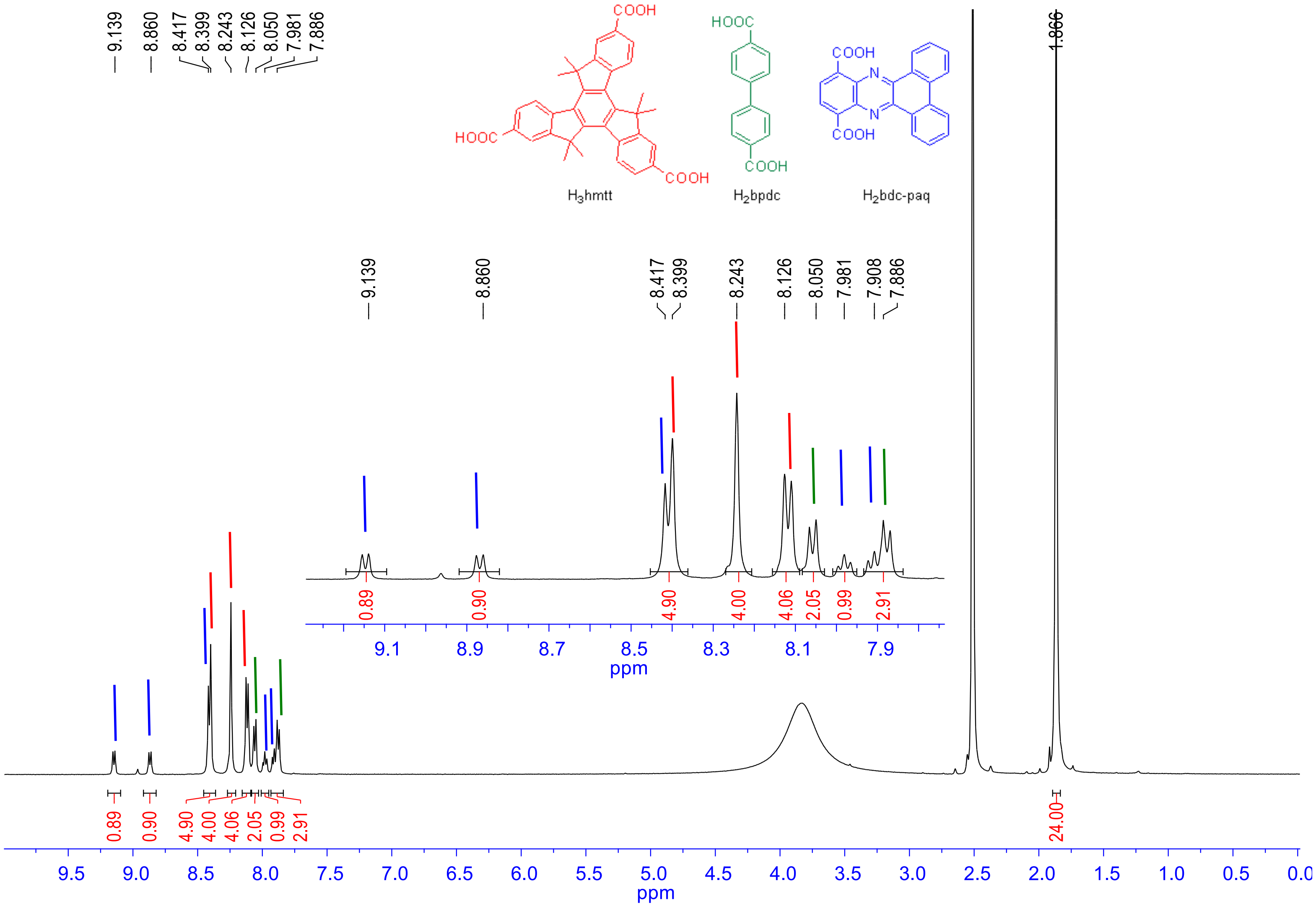

Figure S32: ^{1}H NMR spectrum of digested, NC-MUF-77-bdc-paq**,** $[Zn_4O(hmtt)_{4/3}(bpdc)_{1/2}(bdc\text{-}paq)_{1/2}]$ showing integrals that match with the formula.

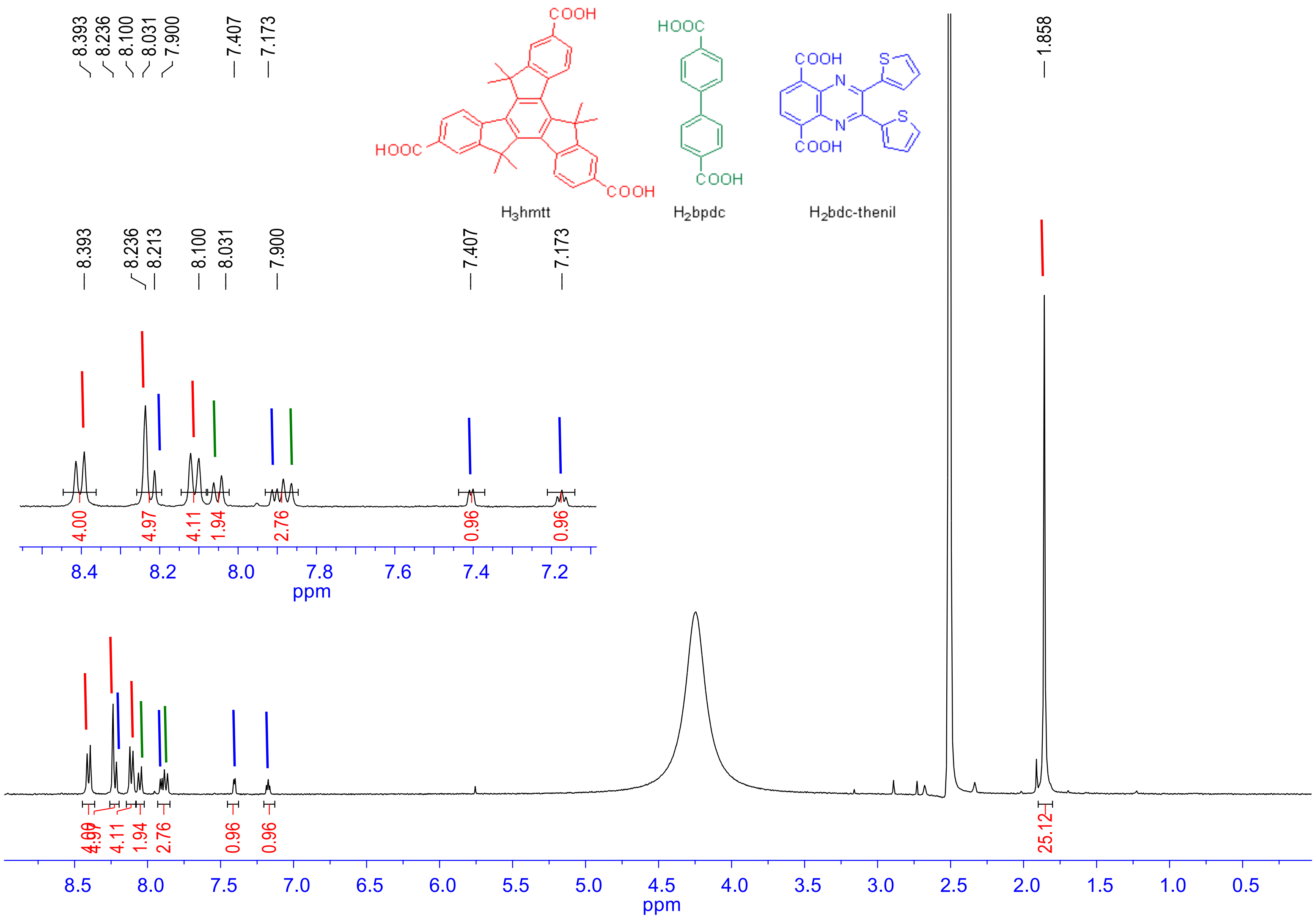

Figure S33: ^{1}H NMR spectrum of digested NC-MUF-77-bdc-thenil, $[Zn_4O(hmtt)_{4/3}(bpdc)_{1/2}(bdc\text{-}thenil)_{1/2}]$ showing integrals that match with the formula.

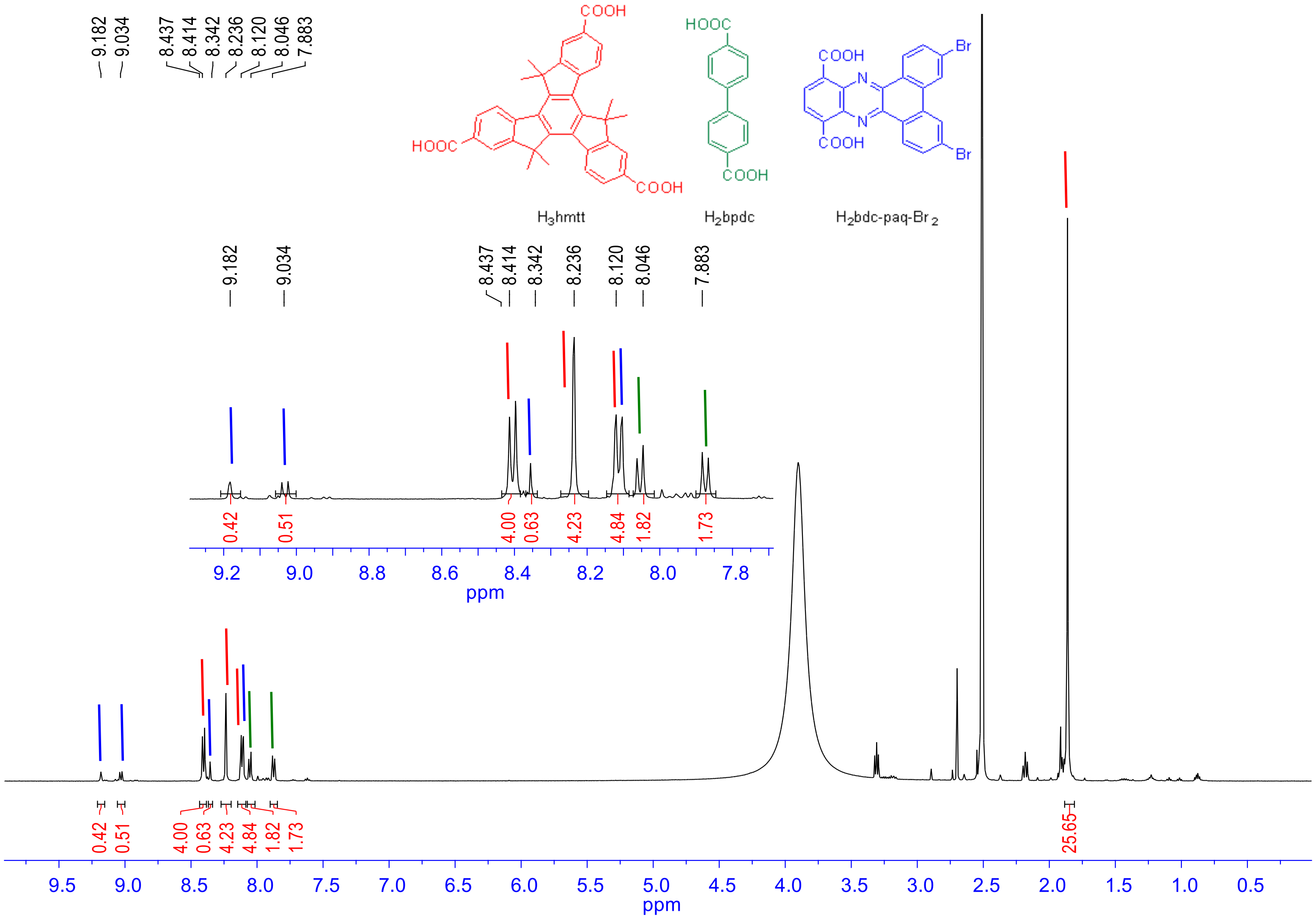

Figure S34: ^{1}H NMR spectrum of digested NC-MUF-77-bdc-paq-Br, [$Zn_4O(hmtt)_{4/3}(bpdc)_{1/2}(bdc\text{-}paq\text{-}Br)_{1/2}$].

18. References

(1) Zhao, Y.; Han, X.; Yu, F.; Wei, D.; Cheng, Q.; Meng, X.; Ding, J.; Hou, H. Direct Conversion of Benzothiadiazole to Benzimidazole: New Benzimidazole-Derived Metal–Organic Frameworks with Adjustable Honeycomb-Like Cavities. *Chem. Eur. J.* **2019,** *25*, 5246.

(2) Conyard, J.; Stacko, P.; Chen, J.; McDonagh, S.; Hall, C. R.; Laptenok, S. P.; Browne, W. R.; Feringa, B. L.; Meech, S. R. Ultrafast Excited State Dynamics in Molecular Motors: Coupling of Motor Length to Medium Viscosity. *J. Phys. Chem. A* **2017,** *121*, 2138.

(3) Eddaoudi, M.; Kim, J.; Rosi, N.; Vodak, D.; Wachter, J.; O'Keeffe, M.; Yaghi, O. M. Systematic design of pore size and functionality in isoreticular MOFs and their application in methane storage. *Science* **2002,** *295*, 469.

(4) Weeks, R. A. Paramagnetic Spectra of E_2' Centers in Crystalline Quartz. *Physical Review* **1963,** *130*, 570.

(5) Sheldrick, G. Crystal structure refinement with SHELXL. *Acta Cryst. C* **2015,** *71*, 3.

(6) Dolomanov, O. V.; Bourhis, L. J.; Gildea, R. J.; Howard, J. A. K.; Puschmann, H. OLEX2: a complete structure solution, refinement and analysis program. *J. Appl. Crystallogr.* **2009,** *42*, 339.
